# Virologic outcomes after early referral of stable HIV-positive adults initiating ART to community-based adherence clubs in Cape Town, South Africa: A randomised controlled trial

**DOI:** 10.1371/journal.pone.0277018

**Published:** 2022-11-15

**Authors:** Jasantha Odayar, Thokozile R. Malaba, Joanna Allerton, Siti Kabanda, David Huang, Cathy Kalombo, Maia Lesosky, Landon Myer

**Affiliations:** 1 Division of Epidemiology & Biostatistics, School of Public Health & Family Medicine, University of Cape Town, Cape Town, South Africa; 2 Provincial Government of the Western Cape, Cape Town, South Africa; International AIDS Vaccine Initiative, UNITED STATES

## Abstract

**Background:**

Differentiated service delivery (DSD) models are recommended for stable people living with HIV on antiretroviral therapy (ART) but there are few rigorous evaluations of patient outcomes.

**Methods:**

Adherence clubs (ACs) are a form of DSD run by community health workers at community venues with 2–4 monthly ART refills and annual nurse assessments). Clinic-based care involves 2-monthly ART refills and 4-monthly nurse/doctor assessments. We compared virologic outcomes in stable adults randomised to ACs at four months post-ART initiation to those randomised to primary health care (PHC) ART clinics through 12 months on ART in Cape Town, South Africa (NCT03199027). We hypothesised that adults randomised to ACs would be more likely to be virally suppressed at 12 months post-ART initiation, versus adults randomised to continued PHC care. We enrolled consecutive adults on ART for 3–5 months who met local DSD [‘adherence clubs’ (AC)] eligibility (clinically stable, VL<400 copies/mL). The primary outcome was VL<400 copies/mL at 12 months on ART.

**Results:**

Between January 2017 and April 2018, 220 adults were randomised (mean age 35 years; 67% female; median ART duration 18 weeks); 85% and 94% of participants randomised to ACs and PHCs attended their first service visit on schedule respectively. By 12 months on ART, 91% and 93% randomised to ACs and PHCs had a VL<400 copies/mL, respectively. In a binomial model adjusted for age, gender, previous ART use and nadir CD4 cell count, there was no evidence of superiority of ACs compared to clinic-based care (RD, -2.42%; 95% CI, -11.23 to 6.38). Findings were consistent when examining the outcome at a threshold of VL <1000 copies/mL.

**Conclusion:**

Stable adults referred to DSDs at 4 months post-ART initiation had comparable virologic outcomes at 12 months on ART versus PHC clinics, with no evidence of superiority. Further research on long-term outcomes is required.

## Introduction

Globally, 21.7 million of the approximately 37 million people living with HIV were accessing antiretroviral therapy (ART) in 2016 [1]. The consolidated guidelines on the use of ART released by the World Health Organization (WHO) in 2016 recommend ART initiation for all people living with HIV, regardless of clinical and immunological status, a “treat all” approach [2]. Health systems thus need to expand ART access in those not on treatment while maintaining engagement in care and viral suppression in patients already on ART.

To manage this increasingly diverse patient population and support ART expansion and maintenance, the WHO recommends implementation of differentiated service delivery (DSD) models [2–4]. Under this framework, patients who are clinically stable on treatment can be considered for referral to models of care which are simplified and adapted to reduce the burden on both patients and the health care system, allowing for more attention to be paid to new patients and those with complex care needs [2]. A number of different DSD models have been developed which vary based on who provides the care (e.g. doctors, nurses or community health workers [CHWs]), location of care (clinic or community), frequency of visits, and services provided (e.g. ART refills, counselling) [5].

One such approach is the Adherence Club (AC) system, which was first piloted in Cape Town in 2007 and has been widely adopted since 2011 [6,7]. Led by community health workers (CHWs), up to 30 adults meet 2–4 monthly for a quick clinical assessment and collection of pre-packed ART [6–9]. Initially facility-based, the model has since been adapted to operate from community venues [10]. Evidence from observational studies has shown high rates of viral suppression [10,11] as well as a reduced risk of loss to follow-up (LTFU) [8,12,13] in patients managed in ACs compared to clinics. However, observational studies of AC effectiveness are prone to selection bias as the decision to refer patients to ACs in routine care settings is made by clinicians, who may refer those who are more likely to adhere to treatment [4,7]^.^ A cluster randomised trial compared patients in ACs at 12 intervention sites to those eligible for ACs but managed at standard of care facilities at 12 control sites in five provinces of South Africa [14]. Patients in ACs had higher retention and comparable viral suppression at one year, but referral to ACs at intervention sites was done as part of routine care and was thus at risk of similar selection bias as observational studies. In a cluster-randomized trial in Zambia, participation in facility-based ACs was associated with a significantly reduced risk of late drug pick-up compared to clinic care, but viral load outcomes were not assessed [15]. A randomised controlled trial (RCT) in South Africa comparing facility and community-based ACs raised some concern about the effectiveness of ACs: rates of loss to care were high overall and were worse in community-based compared to facility-based ACs, but virological outcomes were not assessed and there was no clinic-based care control arm [16].

Studies on effectiveness of ACs have thus provided conflicting results. Data on viral load outcomes in participants receiving care in ACs compared to clinics are limited and there are few individually randomised trials assessing AC effectiveness. To help address this gap, we compared virological outcomes in stable adults randomised to community-based ACs at four months post-ART initiation to those randomised to general primary health care (PHC) ART clinics which are the standard of care (SOC) through 12 months on ART in Cape Town, South Africa. We hypothesised that adults randomised to the ACs would be more likely to be virally suppressed at 12 months post-ART initiation, compared to adults randomised to continued PHC care.

## Materials and methods

### Design and setting

We conducted a pragmatic randomised controlled superiority trial (S1 Table) to compare outcomes in stable adults referred to ACs at four months post-ART initiation to those managed in general PHC ART clinics through 12 months on ART (ClinicalTrials.gov NCT03199027); registration was approved after the start of enrolment due to an administrative error. The primary outcome was HIV viral suppression at 12 months post-ART initiation.

The trial was conducted at a Community Health Centre (CHC) in an informal settlement in Cape Town, South Africa. The CHC is a large primary health care public sector facility which serves a population of approximately 400 000 who are predominantly of low-socioeconomic status [17]. The antenatal HIV prevalence is estimated at 30% [18]. HIV care is provided at the CHC through an ART clinic on the premises and associated ACs which operate off-site at a community centre approximately 1km from the clinic. The ART clinic had >5000 patients retained in care at the end of 2016, of whom approximately half were in ACs [19].

### ART services

#### General adult PHC ART clinic services

General adult patients are initiated on ART at the PHC ART clinic at the CHC. All participants initiated the local first-line ART regimen of tenofovir (300 mg) + emtricitabine (200 mg) + efavirenz (600 mg) as a fixed-dose combination. Participants randomized to the PHC clinic-based care arm received continued care at the clinic, with no club referral prior to 12 months post-initiation. All clinical care and follow-up were provided by government health services and based on local public sector policies. At the PHC ART clinic, patients receive 2–6 monthly clinician review and medication refills (according to clinician discretion). Standard clinic care includes blood tests (viral load [VL] and ART safety bloods as needed) at four- and twelve-months post-ART initiation and annually thereafter. Patients attending the clinic receive a waiting room health promotion and adherence talk. Patients with clinical or psychosocial concerns may be reviewed more frequently or be referred to higher levels of care. Over the December/January holidays, patients receive three months of medication from the pharmacy (from mid-October to mid-November). Patients may send someone to collect their medication (“a buddy”) on their behalf. There is no immediate follow-up for patients who default from clinic attendance, although home visits are done for those who are lost to follow-up (LTFU) every four months, depending on resources.

#### Club care

As part of routine care services, adults attending the general PHC ART clinic are eligible for referral to ACs if they have been on ART for at least six months, are virally suppressed (VL <400 copies/ml) and have no comorbidities requiring regular clinical follow-up. Participants in this trial who were randomized to the AC arm were immediately referred to ACs. All clinical care and follow-up were provided by government health services and based on public sector policies. Each AC includes 25–30 patients and meets for approximately 60 minutes every two months, except over the December/January holiday period when the appointment interval is four months. Clubs are run by CHWs who provide a group health promotion and adherence talk. CHWs also conduct a weight check and symptom screen and dispense pre-packed ART for each club member. A nurse attends an annual visit per club to perform phlebotomy for routine VL monitoring for each patient and attends the subsequent visit to conduct a clinical assessment and check VL results. Patients who have a high VL (>400 copies/ml), are symptomatic and require further clinical assessment, or who miss a club visit and do not collect their medication within five working days of the scheduled appointment are referred back to the ART clinic. Patients in clubs may send a “buddy” (e.g. a partner, friend or relative) to attend alternate non-clinical visits to collect medication.

### Participants and eligibility

HIV-positive adults ≥18 years of age attending their four-month post-ART initiation visit at the PHC ART clinic and not on treatment for tuberculosis (TB) were screened for eligibility. Those who were pregnant, had an intention to relocate out of Cape Town during the study period or had co-morbidities requiring regular clinical follow-up were ineligible for inclusion. Potentially eligible patients who were interested in the study were asked to return in a week to review results of the four-month post-ART initiation VL test and safety bloods conducted by routine services. These results were needed to screen participants as only those with a VL <400 copies/ml are eligible for referral to adherence clubs and a VL <400 copies/mL was thus an eligibility criterion for inclusion in this trial.

### Sources of data

Data were collected through trial measurement visits conducted at enrolment and at four- and eight-months post-enrolment (eight- and twelve- months post-ART initiation, respectively). Trial measurement visits were conducted by trained interviewers at a dedicated research space separate from routine ART services; study visits were at the same location for all participants regardless of the location of ART service visits. At the start of each measurement visit, participants were instructed to not disclose to interviewers their initial trial allocation. Standardized questionnaires were used at study visits to collect information including demographics, past medical history, HIV disclosure and current ART use. In addition, phlebotomy for 5ml of venous blood was conducted at each visit for batched HIV RNA VL testing by the South African National Health Laboratory Services (NHLS) using the Abbott RealTime HIV-1 Assay (Abbott Laboratories, Abbott Park, Illinois, US); this was independent from VL monitoring done as part of routine healthcare services. Participants who missed trial study visits were traced by study fieldworkers working independently of routine healthcare services. Participants found to have defaulted ART care at any time were counselled by study staff and referred for care when required.

### Outcomes

The primary trial outcome was viral suppression (VL <400 copies/mL based on VL testing at trial measurement visit) at 12 months post-ART initiation. The secondary outcome was a VL <1000 copies/mL at 12 months post-ART initiation.

### Sample size calculation and randomisation process

The projected minimum sample size for the trial was 214 participants. This was based on a superiority comparison using 90% power and a two-sided statistical test at α = 0.05. We used 1:1 randomization and aimed to detect an absolute difference in the primary outcome between trial arms of at least 20%, from an expected frequency of 65% in the PHC arm based on routine data, allowing for 10% loss to follow-up.

The randomisation sequence was generated by an independent statistician using STATA 14 (Stata Corporation, College Station, TX, USA). Randomisation was a 1:1 allocation using a dynamic permuted block design, with allocation via sequentially numbered opaque envelopes. These envelopes were stored in a locked and restricted access cabinet and were accessed by the study coordinator once a participant was fully consented and enrolled in the study. The allocation was then conveyed by the study coordinator to a study staff member who would ensure that the referral was made to the allocated site. The data analyst was blinded to study arm until the main trial analysis was complete.

### Statistical analysis

Primary analyses used a modified intention-to-treat population that included all participants with a VL <400 copies/mL at randomisation. For participants who were LTFU, viral load results at the final study visit were imputed as unsuppressed (VL ≥400 copies/mL for the primary outcome and VL ≥1000 copies/mL for the secondary outcome). Per protocol analyses included all participants with a VL <400 copies/mL at randomisation who had at least one visit at the intended service within four months of referral. Intervention effects were examined across *a priori* subgroups including age, gender, previous antiretroviral exposure and nadir CD4 cell count. Additive binomial regression models were used to examine the effect of trial arm on the primary outcome; results are presented as absolute risk differences (RD) with 95% confidence intervals (CI) [20]. Models were adjusted for participant clinical and demographic characteristics which were thought to predict the outcome while not mediating the intervention effect. Covariates which altered the association or appeared independently associated with the outcome were included in the final model. Generalised estimating equations (GEE) under an unstructured working correlation were conducted as a sensitivity analysis to account for clustering of individuals randomised to the same AC. Analyses were conducted using STATA 14 (Stata Corporation, College Station, TX, USA) and R (R Foundation, Vienna, Austria).

### Ethical approvals

Ethical approval for the study was obtained from the Human Subjects Research Ethics Committee of the Faculty of Health Sciences at the University of Cape Town (REF 764/2016). Written informed consent was obtained from all participants prior to enrolment. The authors confirm that all ongoing and related trials for this intervention are registered.

## Results

**Fig 1:**
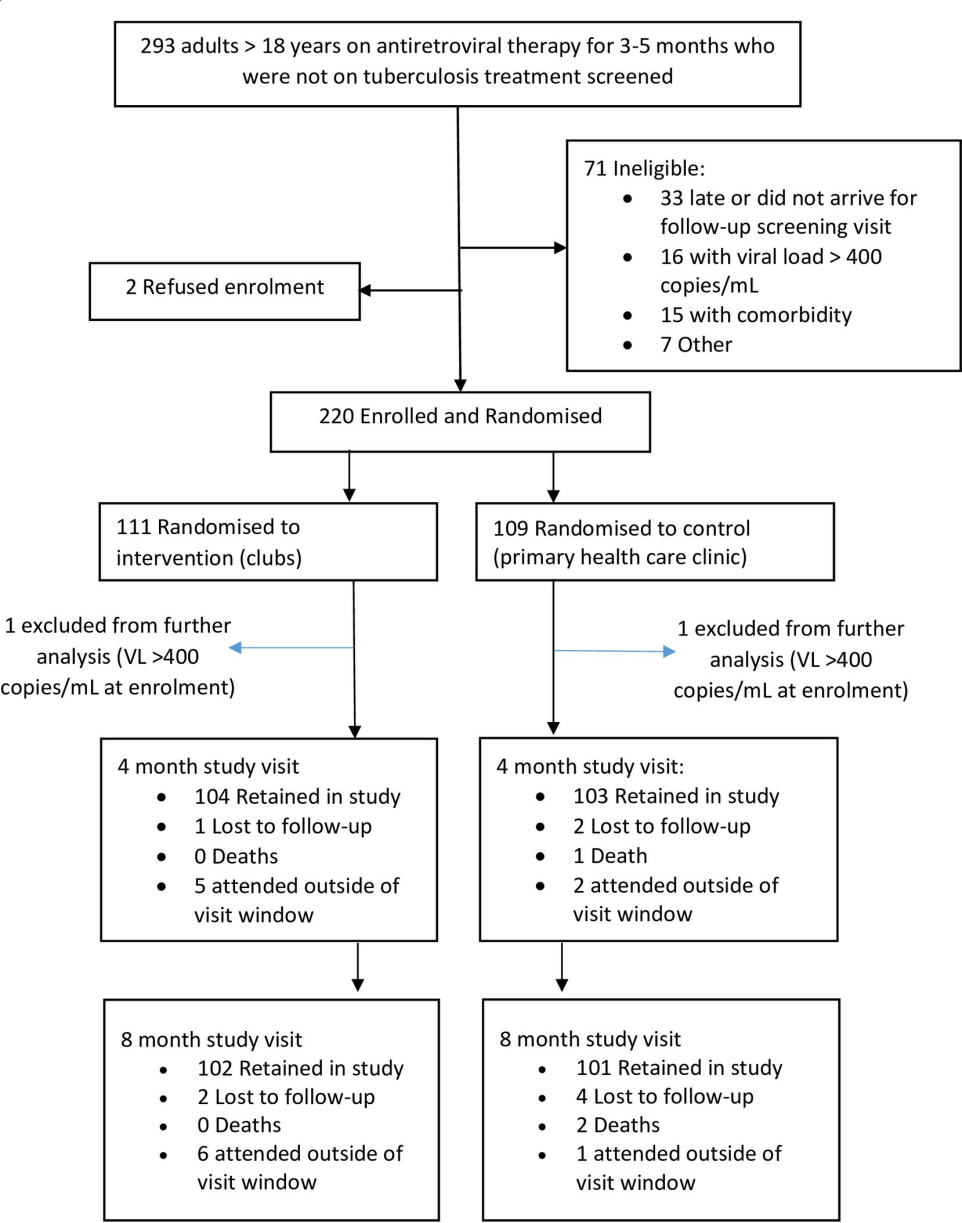
Study enrolment, randomisation and follow-up visits.

Between January 2017 and April 2018, a total of 293 adults who were on ART for 3–5 months and not on TB treatment were screened for inclusion in the trial, of whom 220 were enrolled and randomised (Fig 1). The main reasons for ineligibility were patients not arriving for their follow-up screening visits (n = 33, 47%), VL >400 copies/ml (n = 16, 23%) and the presence of co-morbidities (n = 15, 21%).

Overall, 111 (50.5%) and 109 (49.5%) were randomised to the AC and PHC arms respectively. The VL taken at the first study visit was >400 copies/mL in two participants, one of whom had been randomised to the ACs and one of whom had been randomised to PHC clinics; these participants were excluded from further analysis, leaving 110 participants in the AC arm and 108 in the PHC arm in the final analysis.

Table 1 shows characteristics at enrolment, overall and by trial arm. Overall, median age was 34.7 years, 146 (67%) were female, median nadir CD4 count was 364 cells/μl (IQR, 225–496) and median time on ART was 18 weeks (IQR, 17–20). At this visit, 196 participants (90%) had a VL <100 copies/mL and 22 (10%) had a VL 100–400 copies/mL. The 110 participants in the AC arm were referred to one of 48 clubs. There were no differences in enrolment characteristics by arm.

**Table 1:** Enrolment characteristics of the study sample.

		All (n = 218)	Randomized to PHC clinics (n = 108)	Randomized to ACs (n = 110)
Median age (IQR), years		34.7 (29.1–42.2)	34.5 (28.5–44.0)	35.0 (29.2–42.0)
Gender				
	Female	146 (67.0)	72 (66.7)	74 (67.3)
	Male	72 (33.0)	36 (33.3)	36 (32.7)
Home language: IsiXhosa		199 (91.3)	102 (94.4)	97 (88.2)
Completed secondary/any tertiary education		203 (93.1)	101 (93.5)	102 (92.7)
Currently employed and/or studying		141 (64.7)	73 (67.6)	68 (61.8)
Currently in a relationship		149 (68.4)	69 (63.9)	80 (72.7)
WHO stage				
	1	131 (60.1)	66 (61.1)	65 (59.1)
	2	45 (20.6)	22 (20.4)	23 (20.9)
	3	29 (13.3)	14 (13.0)	15 (13.6)
	4	6 (2.8)	3 (2.8)	3 (2.7)
	Unknown	4 (1.8)	2 (1.9)	2 (1.8)
	Missing	3 (1.4)	1 (0.9)	2 (1.8)
Any previous ARV use		50 (22.9)	25 (23.2)	25 (22.7)
Median time on ART (IQR), weeks		18 (17–20)	18 (17–20)	19 (17–20)
Current ART regimen				
TDF/FTC/EFV		218 (100)	108 (100)	110 (100)
Disclosed to anyone other than a health professional		205 (94.0)	102 (94.4)	103 (93.6)
Missed ART dose reported in previous 30 days		76 (34.9)	39 (36.1)	37 (33.6)
Pre-initiation CD4 count (IQR), cells/μl		364 (225–496)	318 (219–495)	379.5 (257–496)
	Missing	5 (2.3)	3 (2.8)	2 (1.8)
Viral load, copies/mL				
	<100	196 (89.9)	96 (88.9)	100 (90.9)
	100–400	22 (10.1)	12 (11.1)	10 (9.1)

AC: Adherence club, ART: Antiretroviral therapy, ARV: Antiretroviral therapy, EFV: Efavirenz, FTC: Emtricitabine, IQR: Inter-quartile range, PHC: Primary health care, TDF: Tenofovir, WHO: World Health Organization.

All participants were followed up through November 2018 with separate trial measurement visits at four months post-enrolment (eight months post-ART initiation) and at eight months post-enrolment (12 months post-ART initiation and study outcome visit). Of the 218 participants included in the analysis, 207 (95%) attended the four-month trial measurement visit, made up of 103/108 (95%) in the PHC ART clinic arm and 104/110 (95%) in the AC arm. A total of 203/218 (93%) participants attended the study outcome visit, made up of 101/108 (94%) in the PHC ART clinic arm and 102/110 (93%) in the AC arm. Among the 15 participants who did not complete the final study visit at 12 months post-ART initiation, there were two deaths, both of whom had been randomised to the PHC clinic arm. There were no substantial differences in enrolment characteristics between those who did and did not complete the final study follow-up visit (S2 Table).

**Fig 2:**
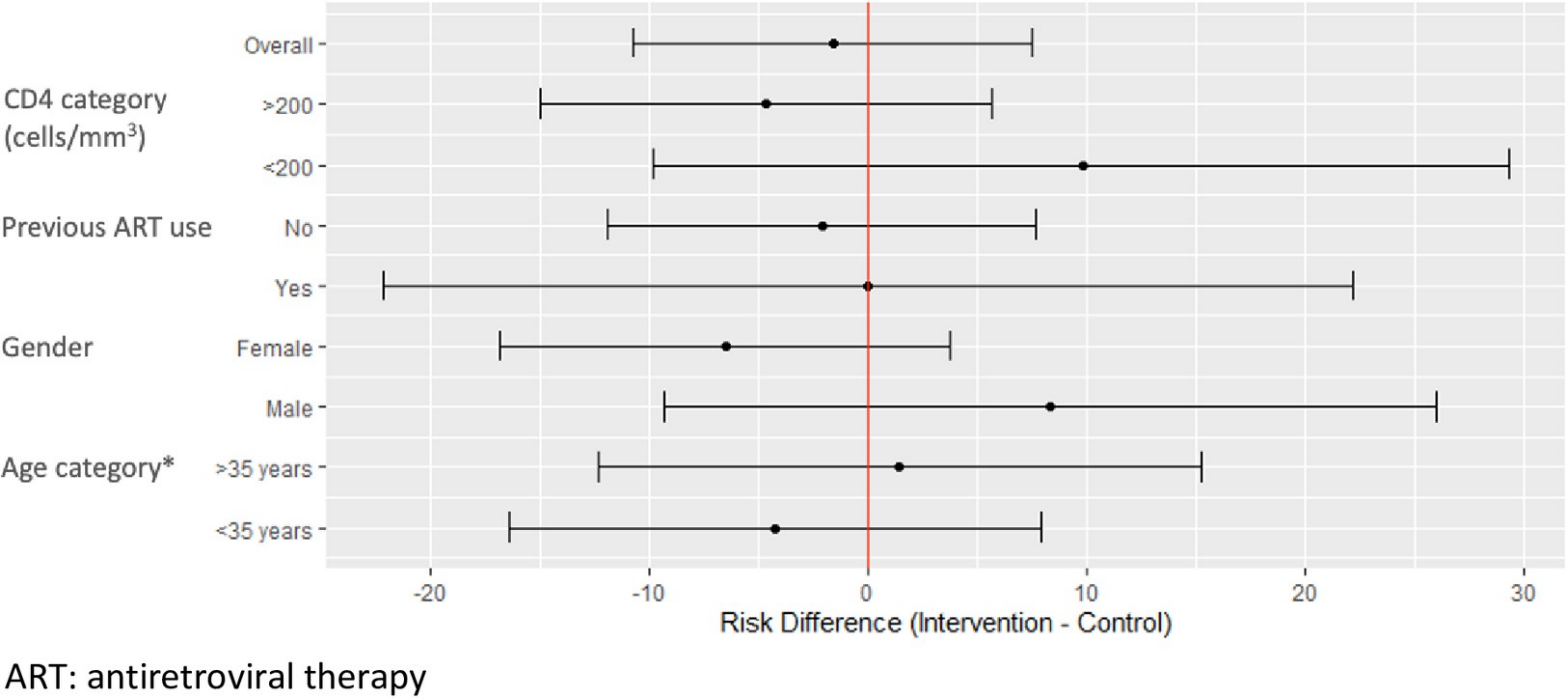
Forest plot of primary outcome (VL <400 copies/mL at 12 months on ART) across a priori subgroups of demographic and clinical characteristics.

Among participants retained at the final study visit, 93% (94/101) of those randomised to the PHC arm and 92% (94/102) of those randomised to the AC arm had a VL<400 copies/mL at 12 months on ART (*p* = 0.816; Table 2). For the MITT analysis, VL results at 12 months on ART were imputed as unsuppressed in those not retained at the final study visit: in this analysis 87% (94/108) of those randomised to the PHC arm and 85% (94/110) of those randomised to the AC arm were categorised as having a VL<400 copies/mL at 12 months on ART (*p* = 0.735). In the 218 participants included in the MITT population, a binomial model adjusted for age, gender, previous ART use and nadir CD4 cell count, found no evidence of superiority of ACs compared to clinic-based care when the outcome was examined at a threshold of VL<400 copies/mL (RD, -2.42%; 95% CI, -11.23 to 6.38; Table 3) and VL<1000 copies/mL (RD, 0.55%; 95% CI, -7.80 to 8.90). In subgroup analyses, males randomised to ACs were more likely to have a VL <400 copies/mL at 12 months on ART compared to males randomised to PHC clinics, while females randomised to ACs were less likely to have a VL <400 copies/mL at 12 months on ART compared to females randomised to PHC clinics but the numbers in subgroup analyses were small and confidence intervals were wide (Table 4 and Fig 2). Adding an interaction term between randomisation allocation and age did not substantially alter estimates but the confidence interval for the difference in risk between those randomised to the AC arm compared to the PHC arm was wide (RD -4.43% [95% CI, -36.56 to 27.69]; Table 5). Clustering by the AC facility to which participants in the intervention arm were referred using GEE (Table 6) produced similar results to the primary analysis.

**Table 2:** Comparison of VL outcomes between trial arms through 12 months post-ART initiation in those who completed the final study visit.

Viral load	Total (n = 203)	Randomised to PHC arm (n = 101)	Randomised to AC arm (n = 102)	*p*-value
VL <400 copies/ml, n (%)	188 (92.6)	94 (93.1)	94 (92.2)	0.816
VL<1000 copies/ml, n (%)	191 (94.1)	94 (93.1)	97 (95.1)	0.567

AC: Adherence club, PHC: Primary health care, VL: Viral load.

**Table 3:** Results of additive binomial model examining the association between trial arm and primary outcome (VL<400 copies/mL) and secondary outcome (VL <1000 copies/mL) adjusted for demographic and clinical characteristics (n = 218).

	Primary outcome (VL <400 copies/mL)		Secondary outcome (VL <1000 copies/mL)	
	Risk difference	95% CI	Risk difference	95% CI
Trial arm (intervention-control)	-2.42%	-11.23 to 6.38	0.55%	-7.80 to 8.90
Gender (male vs female)	-6.02%	-16.31 to 4.27	-3.00%	-12.47 to 6.46
Previous ART use (previous ART use vs no previous ART use)	-7.27%	-19.29 to 4.74	-9.85%	-21.90 to 2.21
Nadir CD4 cell count category (>200 vs ≤200)	-0.02%	-11.00 to 10.97	1.86%	-9.05 to 12.77
Age (years)	-0.009%	-0.44 to 0.42	0.02%	-0.38 to 0.42

ART: Antiretroviral therapy, CI: Confidence interval, VL: Viral load.

**Table 4:** Results of additive binomial models examining the association between trial arm and primary outcome (VL<400 copies/mL) in a priori subgroups of demographic and clinical characteristics (unadjusted).

	Risk difference (intervention-control)	95% CI
Gender Female (n = 146) Male (n = 72)	-6.53% 8.33%	-16.85 to 3.79 -9.33 to 26.00
Previous ART use No (n = 168) Yes (n = 50)	-2.10% -0.001%	-11.88 to 7.68 -22.17 to 22.17
Nadir CD4 cell count category <200 (n = 46) ≥200 (n = 172)	9.85% -4.65%	-9.79 to 29.39 -14.98 to 5.68
Age category <35 years (n = 113) ≥35 years (n = 105)	-4.20% 1.45%	-16.38 to 7.97 -12.33 to 15.24

ART: Antiretroviral therapy, CI: Confidence interval.

**Table 5:** Results of additive binomial model examining the association between trial arm and primary outcome (VL<400 copies/mL) including an interaction term between the intervention and age.

	Risk difference	95% CI
Trial arm (intervention-control)	-4.43%	-36.56 to 27.69
Gender (male vs female)	-6.07%	-16.37 to 4.23
Previous ART use (previous ART use vs no previous ART use)	-7.17%	-19.17 to 4.83
Nadir CD4 cell count category (>200 vs ≤200)	-0.35%	-11.28 to 10.57
Age (years)	-0.09%	-1.37 to 1.19
Interaction: Age (years)*randomisation allocation	0.06%	-0.80 to 0.92

**Table 6:** Generalized estimating equations with additive binomial regression (clustered by adherence club to which participants randomised to the intervention arm were referred) predicting future VL >400 copies/mL.

	Risk difference (%)	95% CI
Trial arm (intervention-control)	-0.14%	-8.41 to 8.14
Gender (male vs female)	-6.42%	-16.70 to 3.85
Previous ART use (previous ART use vs no previous ART use)	-8.47%	-20.69 to 3.76
Nadir CD4 cell count category (>200 vs ≤200)	0.75%	-10.23 to 11.73
Age (years)	-0.007%	-0.43 to 0.41

ART: Antiretroviral therapy, CI: Confidence interval.

Among the 218 participants included in the analysis, 85% (n = 94/110) of those referred to ACs and 94% (102/108) of those referred to PHC ART clinics attended the allocated service within four months of their scheduled visit and were included in the per protocol analysis. S3 Table compares the characteristics of those who did and did not attend the allocated site within four months of their scheduled visit. Among those randomised to ACs, median pre-initiation CD4 count was lower (247.0 cells/μl, IQR 159.5–475.5) in those who did not attend their first visit within four months compared to those who did (399.5 cells/μl, IQR 279.5–496.0). Similarly, in those randomised to the PHC arm, the median pre-initiation CD4 count was lower (209.0 cells/μl, IQR 168.0–307.0) in those who did not attend their first visit within four months compared to those who did (333.0 cells/μl, IQR 224–516). In the per protocol population, there was no significant difference in risk of viral suppression at 12 months in those randomised to the AC arm compared to those randomised to the PHC arm (RD, -2.66%; 95% CI, -11.56 to 6.24) (S4 Table).

## Discussion

To date there have been few randomised studies of DSD models of care for ART delivery. These novel data show no significant difference in virologic outcomes through 12 months post-ART initiation in adults randomised to referral to ACs at four months post-ART initiation versus those who received continued clinic-based care. The sample size here is limited, but the absence of evidence for the superiority of facility-based care over ACs is notable and appears consistent across subgroups of age, gender, previous ART use and CD4 cell count.

This is one of the only RCTs to compare DSD to facility-based services, but previous observational studies have shown marked benefit of ACs compared to routine care. Amogng 2113 patients referred to the same community-based ACs as in this trial, AC participation was associated with a 67% reduction in the risk of LTFU after 12 months compared with attendance at the PHC clinic [8]. Also at a facility in Cape Town, club participation reduced loss-to-care by 57% and reduced virologic rebound in patients who were initially suppressed by 67% [13]. The lack of difference between arms in this trial may be due to randomisation: in observational studies, referral of eligible patients to ACs is based on clinician discretion and patients chosen for referral are likely to be the most adherent [16]. In addition, randomisation may disrupt peer support structures by preventing patients from being referred together. A cluster randomised trial in South Africa which compared facility or community-based ACs to local clinic ART care had findings more in line with ours, with comparable levels of viral suppression found in the two groups [14]. ACs have previously been found to be cost-effective [21], acceptable to patients and health care providers, and there is evidence of sustainability in the Western Cape [12,15,21,22]; considering these factors, a finding of comparable patient outcomes between groups may be considered adequate to support referral of stable patients to ACs in this setting [14]. However, follow-up in this evaluation was through 12 months post-ART initiation; considering that ART is lifelong, an assessment of longer-term outcomes is warranted.

A key finding of this trial is that 6% of those referred to the PHC ART clinic and 15% of those referred to ACs did not attend the allocated service within four months of referral. Data on attendance at facilities other than the specific PHC facility and AC to which participants were referred at randomisation were unavailable. These participants may thus have accessed care at other health facilities or may have been lost to follow-up. A study in postpartum women who initiated ART in pregnancy and were referred to community-based ACs found that 15% of women never attended the allocated service [23]. Among participants randomised to ACs in our trial, those who did not attend the first visit were older and had lower CD4 cell counts than those who did attend, and a higher proportion who did not attend had missed an ART dose in the preceding 30 days. A possible reason for the lower first visit attendance in participants referred to the ACs compared to the PHC clinics is that participants randomised to the PHC clinic arm continued care at the same facility, while participants randomised to the ACs were referred to an off-site community centre. Transfer between health facilities has been identified as a high-risk period for disengagement from care among adults on ART [24]. However, first visit attendance post-transfer from general adult services to DSDs has not been specifically addressed and warrants further investigation, including identification of predictors of first visit non-attendance.

Strengths of these data include the random allocation of participants to either ACs or the PHC clinics, high retention rates and use of an objective outcome measure (VL) which was conducted separate to routine study visits. Limitations include the conduct of the study at a single set of facilities, limiting generalisability. We also did not have data on participant attendance at health care facilities other than the facilities at which the study was conducted. As part of the sample size calculation, we estimated that 65% of individuals randomised to the PHC arm would remain virally suppressed based on VL reporting from the facility at which the study was conducted. However, viral suppression rates in individuals retained in care were higher than expected. In addition, retention in care in both trial arms was high, possibly due to follow-up and tracing activities conducted as part of the trial. The figure of 65% thus proved to be an underestimate, contributing to imprecise results. We further note that subgroup analyses should be interpreted with caution due to small numbers. All participants were on TDF, FTC and EFV which was the first-line ART regimen at the time of the trial. In 2019, the first-line ART regimen in adults in South Africa was changed to TDF, FTC and dolutegravir [25]. Dolutegravir may be more potent than efavirenz [26] and it is possible that the difference between arms would be smaller using the current regimen.

In summary, we found comparable virologic outcomes at 12 months on ART in stable adults referred to ACs versus those who received continued clinic-based care. Further research is required to investigate long-term outcomes. Approximately 15% of those referred to ACs did not attend an AC visit and this warrants further investigation.

## Supporting information

S1 Table CONSORT diagram. (DOCX)

S2 Table Characteristics of participants completing the final study visit through 12 months, versus those not completing the final study visit through 12 months for any reason by randomisation allocation. (DOCX)

S3 Table Characteristics of participants who attended the allocated service within four months of randomisation versus those not attending the allocated service within four months of randomisation by randomisation allocation. (DOCX)

S4 Table Results of additive binomial model examining the association between trial arm and primary outcome (VL<400 copies/mL) in per protocol population adjusted for demographic and clinical characteristics (n = 218). (DOCX)

S1 Protocol (DOCX)

## References

[1] Joint United Nations Programme on HIV/AIDS (UNAIDS). *Miles To Go*. Geneva, 2018.

[2] World Health Organization. *Consolidated Guidelines on the Use of Antiretroviral Drugs for Treating and Preventing HIV Infection*. Geneva, 2016. 27466667

[3] Ehrenkranz P, Grimsrud A, Rabkin M. Differentiated service delivery: navigating the path to scale. Curr Opin HIV AIDS 2019;14:60–5. doi: 10.1097/COH.0000000000000509 30394947

[4] Grimsrud A, Bygrave H, Doherty M, Ehrenkranz P, Ellman T, Ferris R, et al. Reimagining HIV service delivery: the role of differentiated care from prevention to suppression. J Int AIDS Soc 2016;19:21484. doi: 10.7448/IAS.19.1.21484 27914186 PMC5136137

[5] Okere NE, Lennox L, Urlings L, Ford N, Naniche D, Rinke de Wit TF, et al. Exploring Sustainability in the Era of Differentiated HIV Service Delivery in Sub-Saharan Africa: A Systematic Review. J Acquir Immune Defic Syndr 2021;87(4):1055–71. doi: 10.1097/QAI.0000000000002688 33770063 PMC8219088

[6] Wilkinson LS. ART Adherence Clubs: A Long-Term Retention Strategy for Clinically Stable Patients Receiving Antiretroviral Therapy. South Afr J HIV Med 2013;14(2).

[7] Bemelmans M, Baert S, Goemaere E, Wilkinson L, Vandendyck M, van Cutsem G, et al. Community-supported models of care for people on HIV treatment in sub-Saharan Africa. Trop Med Int Heal 2014;19(8):968–77. doi: 10.1111/tmi.12332 24889337

[8] Grimsrud A, Lesosky M, Kalombo C, Bekker L-G, Myer L. Community-Based Adherence Clubs for the Management of Stable Antiretroviral Therapy Patients in Cape Town, South Africa: A Cohort Study. J Acquir Immune Defic Syndr 2016;71(1):e16–23. 26473798 10.1097/QAI.0000000000000863

[9] National Department of Health. *Adherence Guidelines for HIV*, *TB and NCDs*. Pretoria, 2016.

[10] Grimsrud A, Sharp J, Kalombo C, Bekker L-G, Myer L. Implementation of community-based adherence clubs for stable antiretroviral therapy patients in Cape Town, South Africa. J Int AIDS Soc 2015;18(1):19984. doi: 10.7448/IAS.18.1.19984 26022654 PMC4444752

[11] Tsondai PR, Wilkinson LS, Grimsrud A, Mdlalo PT, Ullauri A, Boulle A. High rates of retention and viral suppression in the scale-up of antiretroviral therapy adherence clubs in Cape Town, South Africa. J Int AIDS Soc 2017;20(Suppl 4):21649. doi: 10.7448/IAS.20.5.21649 28770595 PMC5577696

[12] Bock P, Gunst C, Maschilla L, Holtman R, Grobbelaar N, Wademan D, et al. Retention in care and factors critical for effectively implementing antiretroviral adherence clubs in a rural district in South Africa. J Int AIDS Soc 2019;22(10):e25396. doi: 10.1002/jia2.25396 31588668 PMC6778813

[13] Luque-Fernandez MA, Van Cutsem G, Goemaere E, Hilderbrand K, Schomaker M, Mantangana N, et al. Effectiveness of Patient Adherence Groups as a Model of Care for Stable Patients on Antiretroviral Therapy in Khayelitsha, Cape Town, South Africa. PLoS One 2013;8(2):e56088. doi: 10.1371/journal.pone.0056088 23418518 PMC3571960

[14] Fox MP, Pascoe S, Huber AN, Murphy J, Phokojoe M, Gorgens M, et al. Adherence clubs and decentralized medication delivery to support patient retention and sustained viral suppression in care: Results from a cluster-randomized evaluation of differentiated ART delivery models in South Africa. PLoS Med 2019;16(7):e1002874. doi: 10.1371/journal.pmed.1002874 31335865 PMC6650049

[15] Roy M, Bolton-Moore C, Sikazwe I, Mukumbwa-Mwenechanya M, Efronson E, Mwamba C, et al. Participation in adherence clubs and on-time drug pickup among HIV-infected adults in Zambia: A matched-pair cluster randomized trial. PLoS Med 2020;17(7):e1003116. doi: 10.1371/journal.pmed.1003116 32609756 PMC7329062

[16] Hanrahan CF, Schwartz SR, Mudavanhu M, West NS, Mutunga L, Keyser V, et al. The impact of community- versus clinic-based adherence clubs on loss from care and viral suppression for antiretroviral therapy patients: Findings from a pragmatic randomized controlled trial in South Africa. PLoS Med 2019;16(5):e1002808. doi: 10.1371/journal.pmed.1002808 31112543 PMC6528966

[17] Myer L, Phillips TK, Zerbe A, Brittain K, Lesosky M, Hsiao N-Y, et al. Integration of Postpartum Healthcare Services for HIV-Infected Women and their Infants in South Africa: A Randomised Controlled Trial. PLoS Med 2018;15(3):e1002547. doi: 10.1371/journal.pmed.1002547 29601570 PMC5877834

[18] Myer L, Phillips T, Manuelli V, McIntyre J, Bekker L-G, Abrams EJ. Evolution of antiretroviral therapy services for HIV-infected pregnant women in Cape Town, South Africa. J Acquir Immune Defic Syndr 2016;69(2):e57–e65. 10.1097/QAI.0000000000000584 PMC4550573 25723138

[19] Venables E, Towriss C, Rini Z, Nxiba X, Cassidy T, Grimsrud A, et al. Patient experiences of ART adherence clubs in Khayelitsha and Gugulethu, Cape Town, South Africa: A qualitative study. PLoS One 2019;14(6):e0218340. doi: 10.1371/journal.pone.0218340 31220116 PMC6586296

[20] Kovalchik SA, Varadhan R, Fetterman B, Poitras NE, Wacholder S, Katki HA. A general binomial regression model to estimate standardized risk differences from binary response data. Stat Med. 2013; 32(5):808–821. doi: 10.1002/sim.5553 22865328 PMC3982929

[21] Bango F, Ashmore J, Wilkinson L, van Cutsem G, Cleary S. Adherence clubs for long-term provision of antiretroviral therapy: cost-effectiveness and access analysis from Khayelitsha, South Africa. Trop Med Int Heal 2016;21(9):1115–23. doi: 10.1111/tmi.12736 27300077

[22] Dudhia R, Kagee A. Experiences of participating in an antiretroviral treatment adherence club. Psychol Heal Med 2015;20(4):488–94. 10.1080/13548506.2014.953962 PMC4550101 25168720

[23] Myer L, Iyun V, Zerbe A, Phillips TK, Brittain K, Mukonda E, et al. Differentiated models of care for postpartum women on antiretroviral therapy in Cape Town, South Africa: a cohort study. J Int AIDS Soc 2017;20(Suppl 4):21636. doi: 10.7448/IAS.20.5.21636 28770593 PMC5577773

[24] Odayar J, Myer L. Transfer of primary care patients receiving chronic care: the next step in the continuum of care. Int Health 2019;11(6):432–9. doi: 10.1093/inthealth/ihz014 31081907 PMC6854608

[25] South African National Department of Health. *2019 ART Clinical Guidelines*. Pretoria, 2019.

[26] Boffito M, Waters L, Cahn P, Paredes R, Koteff J, Van Wyk J, et al. Perspectives on the Barrier to Resistance for Dolutegravir + Lamivudine, a Two-Drug Antiretroviral Therapy for HIV-1 Infection. AIDS Res Hum Retroviruses 2020;36(1):13–18. doi: 10.1089/AID.2019.0171 31507204 PMC6944139

