# Supplementary material for: Virologic outcomes after early referral of stable HIV-positive adults initiating ART to community-based adherence clubs in Cape Town, South Africa: A randomised controlled trial
Source: PLoS One. 2022 Nov 15;17(11):e0277018. doi: 10.1371/journal.pone.0277018 (PMC9665366; doi:10.1371/journal.pone.0277018)
Supplement: S1 Table — (DOCX) [file pone.0277018.s001.docx]

**Supplementary table 1: CONSORT diagram**

**
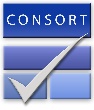
CONSORT 2010 checklist of information to include when reporting a randomised trial***

***Virologic Outcomes After Early Referral of Stable HIV-Positive Adults Initiating ART to Community-Based Adherence Clubs in Cape Town, South Africa: A Randomised Controlled Trial***

| **Section/Topic** | **Item No** | **Checklist item** | **Reported on page No** |
| --- | --- | --- | --- |
| **Title and abstract** | | | |
|  | 1a | Identification as a randomised trial in the title | Title page |
|  | 1b | Structured summary of trial design, methods, results, and conclusions (for specific guidance see CONSORT for abstracts) | On page called “abstract” |
| **Introduction** | | | |
| Background and objectives | 2a | Scientific background and explanation of rationale | Introduction, paragraphs 1-4 |
|  | 2b | Specific objectives or hypotheses | Methods, section titled “Design and setting”, paragraph 1 |
| **Methods** | | | |
| Trial design | 3a | Description of trial design (such as parallel, factorial) including allocation ratio | Methods, Design and setting, para 1 and Methods, section titled “Randomisation” |
|  | 3b | Important changes to methods after trial commencement (such as eligibility criteria), with reasons | NA |
| Participants | 4a | Eligibility criteria for participants | Methods, section titled “Participants and eligibility” |
|  | 4b | Settings and locations where the data were collected | Methods, Design and setting, paragraph 2 |
| Interventions | 5 | The interventions for each group with sufficient details to allow replication, including how and when they were actually administered | Methods, ART services, sections titled “General Adults PHC ART Clinic Services” and “Club Care” |
| Outcomes | 6a | Completely defined pre-specified primary and secondary outcome measures, including how and when they were assessed | Methods, section titled “Sources of Data” and Methods, section titled “Outcomes” |
|  | 6b | Any changes to trial outcomes after the trial commenced, with reasons | NA |
| Sample size | 7a | How sample size was determined | Methods, section titled “Statistical analysis” |
|  | 7b | When applicable, explanation of any interim analyses and stopping guidelines | NA |
| Randomisation: |  |  |  |
| Sequence generation | 8a | Method used to generate the random allocation sequence | Methods, section titled “Randomisation” |
|  | 8b | Type of randomisation; details of any restriction (such as blocking and block size) | Methods, section titled “Randomisation” |
| Allocation concealment mechanism | 9 | Mechanism used to implement the random allocation sequence (such as sequentially numbered containers), describing any steps taken to conceal the sequence until interventions were assigned | Methods, section titled “Randomisation” |
| Implementation | 10 | Who generated the random allocation sequence, who enrolled participants, and who assigned participants to interventions | Methods, section titled “Randomisation” |
| Blinding | 11a | If done, who was blinded after assignment to interventions (for example, participants, care providers, those assessing outcomes) and how | Methods, section titled “Statistical analysis” |
|  | 11b | If relevant, description of the similarity of interventions | NA |
| Statistical methods | 12a | Statistical methods used to compare groups for primary and secondary outcomes | Methods, section titled “Statistical analysis” |
|  | 12b | Methods for additional analyses, such as subgroup analyses and adjusted analyses | Methods, section titled “Statistical analysis” |
| **Results** | | | |
| Participant flow (a diagram is strongly recommended) | 13a | For each group, the numbers of participants who were randomly assigned, received intended treatment, and were analysed for the primary outcome | Results, paragraphs 1 and 5 |
|  | 13b | For each group, losses and exclusions after randomisation, together with reasons | Results, paragraph 1 |
| Recruitment | 14a | Dates defining the periods of recruitment and follow-up | Results, paragraphs 1 and 3 |
|  | 14b | Why the trial ended or was stopped | Results, para 1 |
| Baseline data | 15 | A table showing baseline demographic and clinical characteristics for each group | Table 1 in Results |
| Numbers analysed | 16 | For each group, number of participants (denominator) included in each analysis and whether the analysis was by original assigned groups | Results, paragraphs 3–5 |
| Outcomes and estimation | 17a | For each primary and secondary outcome, results for each group, and the estimated effect size and its precision (such as 95% confidence interval) | Results, paragraphs 3–5 |
|  | 17b | For binary outcomes, presentation of both absolute and relative effect sizes is recommended | Results, paragraph 3 |
| Ancillary analyses | 18 | Results of any other analyses performed, including subgroup analyses and adjusted analyses, distinguishing pre-specified from exploratory | Results, paragraphs 3–5 |
| Harms | 19 | All important harms or unintended effects in each group (for specific guidance see CONSORT for harms) | Results, paragraphs 3 and 5 |
| **Discussion** | | | |
| Limitations | 20 | Trial limitations, addressing sources of potential bias, imprecision, and, if relevant, multiplicity of analyses | Discussion, para 4 |
| Generalisability | 21 | Generalisability (external validity, applicability) of the trial findings | Discussion, para 4 |
| Interpretation | 22 | Interpretation consistent with results, balancing benefits and harms, and considering other relevant evidence | Discussion paras 1–3 |
| **Other information** | | |  |
| Registration | 23 | Registration number and name of trial registry | Methods, Design and setting, para 1 |
| Protocol | 24 | Where the full trial protocol can be accessed, if available | The protocol is available on ClinicalTrials.gov (NCT03199027) |
| Funding | 25 | Sources of funding and other support (such as supply of drugs), role of funders | Section titled “Conflicts of Interest and Source of Funding” |

*We strongly recommend reading this statement in conjunction with the CONSORT 2010 Explanation and Elaboration for important clarifications on all the items. If relevant, we also recommend reading CONSORT extensions for cluster randomised trials, non-inferiority and equivalence trials, non-pharmacological treatments, herbal interventions, and pragmatic trials. Additional extensions are forthcoming: for those and for up to date references relevant to this checklist, see www.consort-statement.org


$\pm80\mu l$
