# Supplementary material for: Virologic outcomes after early referral of stable HIV-positive adults initiating ART to community-based adherence clubs in Cape Town, South Africa: A randomised controlled trial
Source: PLoS One. 2022 Nov 15;17(11):e0277018. doi: 10.1371/journal.pone.0277018 (PMC9665366; doi:10.1371/journal.pone.0277018)
Supplement: S2 Table — (DOCX) [file pone.0277018.s002.docx]

**Supplementary table 2: Characteristics of participants completing the final study visit through 12 months, versus those not completing the final study visit through 12 months for any reason by randomisation allocation**

|  | | Completed final study visit (n=203) | Did not complete final study visit (n=15) | *p*-value |
| --- | --- | --- | --- | --- |
| Median age (IQR), years | | 34.7 (29.1-42.2) | 35.8 (24.5-45.6) | 0.961 |
| Gender, n (%) | |  |  | 0.552 |
|  | Female | 137 (67.5) | 9 (60.0) |  |
|  | Male | 66 (32.5) | 6 (40.0) |  |
| Home language: IsiXhosa, n (%) | | 186 (91.6) | 14 (93.3) | 0.626 |
| Completed secondary/any tertiary education, n (%) | | 189 (93.1) | 14 (93.3) | 1.000 |
| Currently employed | | 124 (61.1) | 7 (46.7) | 0.271 |
| Currently in a relationship | | 140 (69.0) | 9 (60.0) | 0.566 |
| WHO stage | |  |  | 0.200 |
|  | 1 | 124 (62.0) | 7 (46.7) |  |
|  | 2 | 42 (21.0) | 3 (20.0) |  |
|  | 3 | 26 (13.0) | 3 (20.0) |  |
|  | 4 | 5 (2.5) | 1 (6.7) |  |
|  | Unknown | 3 (1.5) | 1 (6.7) |  |
|  | Missing: 3 (1.36) |  |  |  |
| Any previous ARV use | | 46 (22.7) | 4 (26.7) | 0.752 |
| Median time on ART (IQR), weeks | | 18.4 (17.0-20.0) | 17.1 (16.7-19.9) | 0.094 |
|  |  |  |  |  |
| Current ART regimen: TDF/FTC/EFV | | 203 (100.0) | 15 (100.00) | 1.000 |
| Disclosed to anyone other than a health professional | | 191 (94.1) | 14 (93.3) | 1.000 |
| Missed ART dose reported in previous 30 days | | 69 (34.0) | 7 (46.7) | 0.320 |
| Pre-initiation CD4 count (IQR), cells/µl | | 362 (225.0-492) | 426 (225-667) | 0.360 |
|  | Missing | 3 | 0 |  |
| Viral load, copies/mL | |  |  | 0.182 |
|  | <100 | 184 (90.64) | 12 (80.00) |  |
|  | >100 | 19 (9.36) | 3 (20.00) |  |
| Randomised to ACs | | 102 (50.2) | 8 (53.3) | 0.897 |
| AC: Adherence club, ART: antiretroviral therapy, ARV: antiretroviral, EFV: efavirenz, FTC: emtricitabine, IQR: inter-quartile range, TDF: tenofovir, WHO: World Health Organization. | | | | |


$\pm80\mu l$
