# Supplementary material for: Virologic outcomes after early referral of stable HIV-positive adults initiating ART to community-based adherence clubs in Cape Town, South Africa: A randomised controlled trial
Source: PLoS One. 2022 Nov 15;17(11):e0277018. doi: 10.1371/journal.pone.0277018 (PMC9665366; doi:10.1371/journal.pone.0277018)
Supplement: S3 Table — (DOCX) [file pone.0277018.s003.docx]

**Supplementary table 3: Characteristics of participants who attended the allocated service within four months of randomisation versus those not attending the allocated service within four months of randomisation by randomisation allocation**

|  | | Randomized to ACs (n=110) | | Randomized to PHC clinics (n=108) | |
| --- | --- | --- | --- | --- | --- |
|  | | **Attended AC within 4 months of randomization (n=94)** | **Did not attend AC within 4 months of randomization (n=16)** | **Attended PHC clinic within 4 months of randomization (n=102)** | **Did not attend PHC clinic within 4 months of randomization (n=6)** |
| Median age (IQR), years | | 35.1 (29.8-42.1) | 33.1 (25.0-37.0) | 34.5 (28.6-44.0) | 36.6 (28.2-44.5) |
| Gender, n (%) | |  |  |  |  |
|  | Female | 65 (69.15) | 9 (56.25) | 68 (66.67) | 4 (66.67) |
|  | Male | 29 (30.85) | 7 (43.75) | 34 (33.33) | 2 (33.33) |
| Home language: IsiXhosa, n (%) | | 82 (87.23) | 15 (93.75) | 97 (95.10) | 5 (83.33) |
| Completed secondary/any tertiary education, n (%) | | 87 (92.55) | 15 (93.75) | 97 (95.10) | 4 (66.67) |
| Currently employed | | 60 (63.83) | 8 (50.00) | 68 (66.67) | 5 (83.33) |
| Currently in a relationship | | 67 (71.28) | 13 (81.25) | 65 (63.7) | 4 (66.67) |
| WHO stage | |  |  |  |  |
|  | 1 | 58 (61.70) | 7 (43.75) | 61 (59.80) | 5 (83.3) |
|  | 2 | 16 (17.02) | 7 (43.75) | 21 (20.59) | 1 (16.67) |
|  | 3 | 14 (14.89) | 1 (6.25) | 14 (13.73) | 0 |
|  | 4 | 2 (2.13) | 1 (6.25) | 3 (2.94) | 0 |
|  | Unknown | 2 (2.13) | 0 | 2 (1.96) | 0 |
|  |  | 2 (2.13) | 0 | 1 (0.98) | 0 |
|  |  |  |  |  |  |
| Any previous ARV use | | 22 (23.40) | 3 (18.75) | 24 (23.53) | 1 (16.67) |
| Median time on ART (IQR), weeks | | 18.4 (17.0-20.0) | 19.5 (17.9-20.3) | 18.1 (17.0-20.0) | 18.0 (17.3-19.3) |
|  |  |  |  |  |  |
| Current ART regimen: TDF/FTC/EFV | | 88 (100.00) | 15 (100.00) | 98 (100.00) | 4 (100.00) |
| Disclosed to anyone other than a health professional | | 87 (92.55) | 16 (100.00) | 97 (95.10) | 5 (83.3) |
| Missed ART dose reported in previous 30 days | | 30 (31.91) | 7 (43.75) | 37 (36.27) | 2 (33.33) |
| Pre-initiation CD4 count (IQR), cells/µl | | 399.5 (279.5-496) | 247 (159.5-475.5) | 333 (224-516) | 209 (168-307) |
|  | Missing: 5 | 2 (2.13) | 0 | 3 (2.94) | 0 |
| Viral load, copies/mL | |  |  |  |  |
|  | <100 | 85 (90.43) | 15 (93.75) | 91 (89.22) | 5 (83.33) |
|  | >100 | 9 (9.57) | 1 (6.25) | 11 (10.78) | 1 (16.67) |
| AC: Adherence club, ART: antiretroviral therapy, ARV: antiretroviral, EFV: efavirenz, FTC: Emtricitabine, IQR: inter-quartile range, PHC: primary health care, TDF: tenofovir. | | | | | |


$\pm80\mu l$
