# Supplementary material for: Virologic outcomes after early referral of stable HIV-positive adults initiating ART to community-based adherence clubs in Cape Town, South Africa: A randomised controlled trial
Source: PLoS One. 2022 Nov 15;17(11):e0277018. doi: 10.1371/journal.pone.0277018 (PMC9665366; doi:10.1371/journal.pone.0277018)
Supplement: S4 Table — (DOCX) [file pone.0277018.s004.docx]

**Supplementary table 4: Results of additive binomial model examining the association between trial arm and primary outcome (VL<400 copies/mL) in per protocol population adjusted for demographic and clinical characteristics (n=196)**

|  | Risk difference | 95% CI |
| --- | --- | --- |
| Trial arm (intervention-control) | -2.66% | -11.56 to 6.24 |
| Sex (male vs female) | -3.26 | -13.46 to 6.94 |
| Previous ART use (previous ART use vs no previous ART use) | -10.49% | -23.30 to 2.33 |
| CD4 category (>200 vs <200) | -1.23 | -12.23 to 9.77 |
| Age (years) | -0.09% | -0.52% to 0.35 |
| ART: antiretroviral therapy, CI: confidence interval. | | |


$\pm80\mu l$
