# Supplementary material for: Virologic outcomes after early referral of stable HIV-positive adults initiating ART to community-based adherence clubs in Cape Town, South Africa: A randomised controlled trial
Source: PLoS One. 2022 Nov 15;17(11):e0277018. doi: 10.1371/journal.pone.0277018 (PMC9665366; doi:10.1371/journal.pone.0277018)
Supplement: S1 Protocol — (DOCX) [file pone.0277018.s005.docx]

**Timing of Referral to Adherence Clubs (TRAC)**

**A sub-study of the PACART trial**

**Version 3**

**Principal Investigator**

Professor Landon Myer

Head of the Division of Epidemiology and Biostatistics

Director: Centre for Infectious Diseases Epidemiology & Research

School of Public Health and Family Medicine

University of Cape Town

**Co-Investigators**

Dr Joanna Allerton

Division of Epidemiology and Biostatistics

School of Public Health and Family Medicine

University of Cape Town

Dr Jasantha Odayar

Division of Epidemiology and Biostatistics

School of Public Health and Family Medicine

University of Cape Town

**1. Background**

In 2014, UNAIDS (Joint United Nations Programme on HIV/AIDS) announced the global “90-90-90” strategy, the target being that by 2020, 90% of people living with HIV will have been diagnosed, 90% of these will be on antiretroviral therapy (ART) and 90% of those on ART will have a suppressed viral load.^1^ In South Africa, however, there are estimated to be 6.47 million people living with HIV, with only 53% of these being engaged in care and 26% of those living with HIV being virally suppressed.^2^ Furthermore, retention at 3 years post ART-initiation in sub-Saharan Africa has been estimated at 65%-70%.^3^ As a suppressed viral load depends primarily on adherence, there is a huge need for effective means of retaining large numbers of people taking ART in care, thereby hoping to increase their adherence and suppress their viral load.^4^ One model of community-based adherence support is the ART adherence club. These were locally piloted from 2007 with the aim of assessing whether this group-based, lay-counsellor led service, with an emphasis on social support and adherence, could help address the needs described particularly with regard to retention in care and viral suppression.^5^,^6^ Since the initial pilots, the club model has been scaled up rapidly with more than 400 clubs meeting in the Cape Town metro.^7^ As this model is being scaled-up and implemented rapidly, there is an urgent necessity to further assess its effectiveness.

A large multi-centre cohort study in South Africa found that community-based adherence support in ART programs improved patient attrition (hazard ratio 0.68, 95% CI=0.65-0.72) compared to no community-based adherence support and concluded that community-based programs should be considered for scale-up.^8^ A more recent systematic review, conducted on low- to middle-income countries found that community-based interventions were at least comparable to facility-based interventions in terms of ART adherence and viral suppression.^9^ Although encouraging results, these studies are not locally or adherence club specific.

Locally, implementation of these clubs has been found to be generally successful and popular with clinic staff and patients, with one local study describing a period of 18 months during which a third of clinic patients were de-centralized to clubs.^10^ Overall ART retention in this study was found to be 97.2% at 6 months (95% CI=95.4-97.8) and 93.5% (95%CI=92.2-94.5) at 12 months, and after a year in a club more than 98% of patients remained virally suppressed.^10^ Although there was no suitable comparison group in this study, these retention rates were shown to be well above the estimated retention rates described above and so this study supported continued expansion of the clubs model.

Analytical studies have shown that local adherence clubs decrease both loss-to-follow-up (hazard ratio 0.43, 95% CI=0.21-0.91) and viral rebound (hazard ratio 0.33, 95% CI=0.16-0.67) compared with usual clinic care.^5^ A later study confirmed this association, finding a loss-to-follow-up hazard ratio of 0.33 (95% CI=0.27-0.40) when comparing club care with clinic care.^11^ These comparative studies add more support in favour of the roll-out of the club model.

Adherence clubs have also been shown to be more cost-effective than clinic care, in low- and middle-income countries^9^ and also locally, with one study estimating a cost per patient-year of $300 for clubs vs $374 for clinic care.^12^ Interviewed club patients reported a higher acceptability of club compared to clinic as well as shorter waiting times.^12^ Again this study concluded by supporting scale-up.

So whilst adherence clubs have been shown to be locally implementable, popular (with buy-in by clinic staff and patients) and cost-effective, and community-based interventions have been shown to improve retention, evidence of local adherence club effectiveness in improving viral suppression and retention has only been observationally obtained.^6^ This observational evidence is highly subject to selection bias, with already-adherent patients being over-represented in the club system because of the club eligibility criteria, which includes a suppressed viral load. Even if this selection bias is limited by including viral suppression as a study eligibility criteria, there still remains the possibility of residual confounding in patient factors such as an openness to club-based care and more inherent adherence.^5^

This trial aims to reduce the effect of selection bias and confounders by using a randomised controlled trial design with two arms – those ART patients receiving care in clinic and those receiving care in clubs. The current local standard of care is clinic-based with potential referral to clubs after 6 months on ART if eligible (eligibility criteria differs slightly facility to facility). In this trial we aim to assess the effectiveness of clubs in achieving retention and a suppressed viral load, amongst other outcomes, by comparing early club referral (at 4 months post-ART initiation) and delayed club referral (after 12 months post-ART initiation). By doing this we hope to generate robust evidence regarding both the effectiveness of clubs and the optimal timing of club referral.

**2. Study Aims and Objectives**

The overall aim of this project is to evaluate the adherence club model compared to the clinic model of care. The two arms of the study will be early referral to the adherence club service (referral by study at 4 months post-initiation) and delayed referral to clubs (after 12 months post-initiation, referral to be done as per usual procedures).

- Primary objective

To compare adult HIV viral suppression up to 12 months post ART-initiation, among adults on ART with a suppressed viral load at 4 months, in those referred to the adherence club at 4 months, compared to those referred to the club after 12 months post-initiation.

- Secondary objectives

To compare the timing of entry into ART clubs, looking at outcomes such as:

- Retention in care ie missed visits and loss to follow-up
- Physical and mental health
- Acceptability of the ART service

This trial is a sub-study of the UCT PACART trial currently underway and as such the setting, population, study design, data management, ethical considerations and internal monitoring systems are designed to be in line with the PACART protocol as much as possible.

**3. Study Design**

Overview

We propose to address these objectives by conducting a pragmatic, two-arm randomised controlled trial to evaluate two different entry points into ART clubs (hereafter referred to as clubs) – at 4 months post-initiation of ART (early) and after 12 months post-initiation of ART (delayed). All adults have a routine clinic visit 4 months post-initiation of ART, for a clinical check-up and viral load testing. Adults attending this visit will be approached by a study recruiter to participate in the trial. If interested in the study and potentially eligible (with the exception of the as yet unknown viral load result), a return visit 1 week later will be arranged to see a member of study staff who will check for a suppressed viral load (<400) and complete enrolment procedures. A total of 214 consenting adults will be randomised to either:

1. Early referral to clubs (at 4 months post-initiation)
2. Delayed referral to clubs (after 12 months post-initiation)

Participants in both arms will be asked to attend 3 study measurement visits in total: at enrolment, and then at4 months and 8 months post-enrolment. These visits will be conducted separately from ART service appointments. Details of study measurement visits are provided in section 7 below. Follow-up of outcomes will be through to 8 months post-enrolment. Participation will impact only on the setting and timing of ART services and will not affect access to care. See below for the study schema:

HIV-positive adults on ART seen at 4 months post-ART initiation at the ART clinic, Gugulethu

**Screening and Informed Consent**

Enrolment of eligible adults, questionnaire administration and phlebotomy for viral load testing

**Randomisation**

**Delayed referral**

Referral to ART clubs after 12 months post-initiation

**Early referral**

Referral to ART clubs at 4 months post-initiation

Setting

The proposed trial will take place at Gugulethu ART clinic in Cape Town and at the associated off-site ART clubs. HIV prevention and treatment services at these facilities have been supported by the University of Cape Town since 2001, and UCT has a dedicated research space for trials on the Gugulethu CHC premises.

Gugulethu has a population of approximately 400000 and is predominantly of low-socioeconomic status. The vast majority of the population uses free local public sector health services. By the end of July 2016, approximately 5259 adults on ART were under the care of the ART services at the Gugulethu Community Health Centre (from which the clinics and clubs are run), with approximately 54% of these being retained in care at the ART clubs (from local data). Since the implementation of clubs at Gugulethu in June 2012, the number of adults retained in club care is steadily increasing. From January 2014 until August 2016, the number of adults retained in club care increased from 2420 to 2850 (from local data).

**4. Study Population**

Participants will be consecutive consenting HIV-positive adults on ART who are seeking care at the Gugulethu ART clinic.

Inclusion criteria

- Age 18 years or older
- Documented HIV infection with ART initiation 4 months ago
- Suppressed viral load at 4 months post-initiation (<400 copies/ml)
- All other month 4 blood results within normal limits
- Willingness to be randomised and return for study measurement visits
- Able and willing to attend service visits at either the clinic or a club
- Able to provide informed consent for research

Exclusion criteria

- Intention to relocate out of Cape Town permanently during the study period
- Any medical, psychiatric or social condition which in the opinion of the investigators would affect the ability to consent and/or participate in the study
- Pregnant
- Current co-morbidity requiring additional health care, either acutely eg tuberculosis or chronically eg hypertension

**5. Recruitment and Enrolment**

Recruitment

This will take place at the Gugulethu ART clinic. Consecutive potentially eligible adults will be approached during their routine 4-month post ART initiation clinic visit, which consists of viral load testing, either directly by the study recruiter or via the attending clinician (who will refer to the study recruiter subject to patient agreement). The potential participant will be given information about the study. If interested and eligible (with the exception of the as-yet unknown viral blood result) they will be given a study visit 1 week later to receive their viral load results from a member of study staff, conclude enrolment procedures and undergo randomisation. At the point of leaving the clinic after their blood test, the potential participant should have the following in place:

- A study visit date for 1 weeks’ time
- A clinic date for 1 months’ time (as per clinic standard of care)
- 1 month of ART (as per clinic standard of care)
- Having had their month 4 blood test, consisting of viral load and safety bloods (as per clinic standard of care)
- Having seen a clinician (as per clinic standard of care)

Enrolment

Enrolment will take place at this follow-up visit (1 week after the month 4 clinical visit), if eligible and consenting. The individual will be informed of his/her viral load result by study staff and, if the viral load is less than 400 copies/ml, he or she will be further screened for eligibility as per the inclusion and exclusion criteria described above. Adults who are deemed eligible and agree to participate will undertake the informed consent process, which will be conducted in a private room by a study interviewer.

Informed consent

Informed consent procedures are outlined in Section 10, below. Trained study staff will ensure that individuals are aware of their right to refuse to participate in the study or to withdraw from the study at any time once enrolled. In addition, study staff will emphasize that all study activities are entirely separate from routine ART care services and that refusal to participate in or withdrawal from the study will have no effect on access to any services provided at public sector health facilities. Moreover, staff will be sure participants understand that their access to ART and any other aspect of their care, will not be affected by their decision to participate. Following, informed consent procedures, enrolled participants will be randomized to either early club referral or delayed club referral.

Recruitment will take place over a 12-18 month period.

**6. The standard ART Services at Gugulethu CHC – the clinic and the clubs**

The local standard of care is clinic-based from ART-initiation, with potential club referral from 6 months post-initiation for those patients who are virally suppressed and have no co-morbidities that require additional health care. Club referral is at the clinician’s discretion. The two models of care are described below:

Clinic care

The standard ART clinic service includes 2-6 monthly clinician reviews and medication refills (according to clinician discretion). Over the December/January holidays, patients receive 3 months of medication from the pharmacy (from mid-October to mid-November). Patients may send someone to collect their medication (“a buddy”) on their behalf. Patients with particular clinical or psychosocial concerns may be reviewed more regularly, or referred to higher levels of care. Patients in the clinic receive a waiting room health promotion and adherence talk (held daily). Standard clinic care includes blood tests at month 12 post-initiation (viral load and ART safety bloods as needed). There is no immediate follow-up for clinic defaulters, although every 4 months a list of those lost to follow-up is generated via the eKapa computer system, and those on the list will be visited at home depending on resources.

Club care

There are currently 127 Adherence clubs operating at Gugulethu CHC. They meet in the nearby off-site community hall iKhwezi. Each club meets 2 monthly with the exception of the December/January holidays whereby the appointment interval is 4 months. The clubs are run by club counsellors who give a health promotion and adherence talk, promote condom use, weigh and check symptoms for each club member and dispense 2 months of pre-packed ART (4 months over the December/January holiday period). Each club receives an annual blood test visit at which point a nurse practitioner performs routine phlebotomy. The following visit is a clinical visit, at which the nurse practitioner checks the blood results and performs a clinical assessment. Patients in clubs may send someone (”a buddy”) to collect their ARTs for them, but only every alternate visit and never on a blood or clinical visit. If a participant misses a club visit, there is a grace period of 5 working days after the scheduled session for them to collect their medication. Those who have not collected their medication within this period are identified as defaulters. These defaulters are followed up by the counsellors via phone calls and home visits, depending on resources, and are removed from club. Patients are also removed from club if they have a high viral load (>400 copies/ml), are pregnant, or have any symptoms meriting further clinical assessment.

A comparison of clinic and club care is tabulated below:

| **AREAS OF CARE** | **MODEL OF CARE** (August 2016) |  |
| --- | --- | --- |
|  | **CLINIC** | **CLUB** |
| **Summary of model** | Seen in the Gugulethu ART clinic | Seen in an offsite hall, iKhwezi, as part of a club |
| **Usual eligibility criteria** | None – all patients on ARTs may attend | >6 months on ART, viral load<400, no other co-morbidities |
| **First appointment date** | Patients with a referral letter can present on any day. Date does not have to be booked with clinic | Patients referred to club must present to the club office, which is located at the clinic, with their clinic folder and referral letter. A counsellor then provides a date for the first visit, slotting the patient into a club with space available. |
| **Attending staff** | Clinician - CNP or doctor | Counsellor. CNP twice a year (blood visit and clinical visit) |
| **Prescription frequency** | 1-6 monthly, depending on clinician discretion | 2 monthly |
| **Christmas holiday arrangments** | Pharmacy dispenses 3 months of ART from mid-October | Pharmacy dispenses 4 months of ART from the beginning of October. |
| **Buddy collections** | Buddies are unmonitored and are able to collect medication on behalf of the patient except when a clinician specifically requests to see the patient. | Buddies are able to collect medication on behalf of the patient on alternate visits, but blood and clinical visits must be attended by the patient. |
| **Counselling (lifestyle and adherence)** | Daily waiting room counsellor talk. Selected (high risk for non-adherence) patients receive pill checks and further counselling | Every club visit |
| **Blood tests** | CD4 annually only if CD4<200 | CD4 annually only if CD4<200 |
|  | Viral load (VL) at month 4, 12 and annually if on first-line regimen.  VL at month 6, 12 and annually if on second-line regimen.  If on tenofovir, creatinine is checked at month 1, 4, 12 and then annually.  If on zidovudine, Hb and differential white cell count is taken at month 1, 2, 3 and 6.  If on aluvia, cholesterol and triglyceride checked at month 3 and yearly if indicated. | VL annually, timing depends on when club blood visit scheduled.  If on tenofovir, creatinine is checked annually.  If on aluvia, cholesterol and triglyceride checked annually if indicated. |
| **Time spent at the location** | Varies from around 2 hours to the whole day. | 1-2 hours. Max around 3 hours for a clinical visit. |
| **Non-attenders follow-up** | Every 4 months, those patients who have not had an ART refill are visited at home by CCWs | Those who don't collect their medication within the 5 day grace period are called and visited (if resources allow) |
| **Procedure for VL 400-1000** | Repeat VL after 6 months | Referred back to clinic, then usual clinic protocols followed |
| **Procedure for VL >1000** | Refer to ROTF service | Referred back to clinic, then usual clinic protocols followed |

**7. Research Procedures**

Randomisation

Randomisation will be performed once enrolment is completed. Randomization will be a 1:1 allocation using a dynamic permuted block design generated by an independent statistician. Randomization numbers will be generated prior to the start of the study, and placed in sequentially numbered opaque envelopes. Randomization envelopes will be stored in a locked and restricted access cabinet in the research clinic that will be accessed by the study coordinator when a participant is fully consented and has been enrolled in the study. There will be independent documentation of the participant identification number, randomization date, and randomization assignment. The study coordinator involved in randomization will not be involved in interviewing participants. The allocation to either early or delayed adherence club referral will be conveyed to both the participant and to the study recruiter.

Early referral arm

The participant will be escorted by study staff straight from enrolment to the club office for referral to the club service. Here, a club counsellor will educate them about the club system and rules, enrol them into a club and give them a club appointment date within 2 months. If more ART will be needed before the date is reached, a study clinician will dispense. The specific club group to which the individual is assigned will depend on space availability within groups. Participants will be allocated to groups non-purposively, and there will be no attempt to cluster participants within a single group. Their clinic appointment, in 1 months’ time for viral load results, will be cancelled.

Delayed referral arm

Individuals randomized to this arm will be seen at the clinic, with no club referral to be done before 12 months post-initiation. After 12 months has elapsed after initiation, the participant will be subject to usual clinic procedures which may include referral to clubs, dependent on the attending clinician as per standard of care.

The participant’s folder will be labelled with a sticker, signifying no referral to the club service to be done by the clinic staff. Those allocated to the delayed referral arm will also have their names entered onto a list to be held by the senior club counsellor and kept in the club office. This list will be checked when participants are referred to the club office and any participants on this list will not be given a club date and will be referred back to the attending clinician

Study visits

Including enrolment (study visit 1), there will be 3 study visits. These will be at enrolment and then at4 months and 8 months post-enrolment.

Measurements will be carried out by a study measurement team comprised of a research nurse and interviewers who operate separately from the routine ART services (adherence clubs and clinic). Visits will take place in a dedicated research space. The separation of routine services from study services will facilitate masking of trial assessments and retention activities.

At each study visit (estimated to last 1-2 hours), a standardized questionnaire will be used by the study interviewers to collect information and phlebotomy will be performed.

**At the enrolment visit and all subsequent study visits**, information will be collected on: demographics, past medical history and health care use, HIV disclosure, current ART use, relationship with health providers, mental health, alcohol and substance use, and social impact.

**Phlebotomy** will involve drawing 5ml of venous blood for storage and batched viral load testing. This same blood will be used (via pipetting from the PPT blood tube) for Dried Blood Spots (DBS). This pipetting method will be used to avoid a further procedure and its incumbent risks.

Laboratory measures

Participant venous blood (approximately 5ml at up to 3 study visits, totalling a maximum of 15ml over 8 months) will be drawn (using an PPT tube) at every study measurement visit. As previously described, some of this blood will be stored as a Dried Blood Spot, for use as an objective marker of adherence. The PPT tube specimen will be transported to the National Health Laboratory Service (NHLS) for testing.

The venous blood collected will be used for HIV viral load batch testing (Abbott Molecular RealTime HIV-1 assay, Abbott Molecular, Illinois, USA). Following VL testing the remaining plasma will be stored as single aliquots of $\pm80\mu l$ in Sarstedt screw cap tubes in a -80°C freezer at the School of Public Health and Family Medicine, UCT. The DBS cards will be transported for storage at -80 °C (subject to consent). These stored specimens will be for future research use.

All phlebotomy, specimen handling, specimen processing, and specimen storage will take place using routine protocols. Note that the laboratory measures taken for study measurements are in addition to routine laboratory tests taken for HIV care and treatment.

Data abstraction

In addition to the study measures described above, routine clinical care record reviews will be conducted in order to collect key data on participants regarding ART initiation and follow-up. Permission to review clinical records of participants, is included in informed consent documents for all participants. The records used will be those at the clinic and club as needed.

Items to be abstracted include: date of HIV diagnosis, date of initial presentation to the ART service, pre-ART clinical history, examination and laboratory investigations (including TB-related history and WHO staging), date of ART initiation, dates of routine follow-up visits attended post-ART initiation, major clinical events post-ART initiation and ART side-effects.

All data abstraction will take place with the written permission of (a) the participant (via informed consent), (b) the research oversight body of the Government of the Western Cape (GWC), (c) the participating REC, and (d) the facility manager of the health facility. Data abstraction will be conducted by trained staff working under the close supervision of the project coordinator. All data will be abstracted from the clinical record onto separate forms that include record of the type of documentation, the date of abstraction, and the name of the individual abstracting data.

All data collection is confidential, with records identified via participant folder numbers only. No participant names are recorded on study documents.

In addition to this data abstraction, permission to review clinical records of participants, including those at government clinics and hospitals within the Western Cape for up to 3 years after enrolment, is included in informed consent documents for all participants.

The health data will be aes-encrypted and password protected, and will be transferred using a secure file transfer service such as filesender. Identifying information and clinical information will be stored and transferred in separate files.

Identification of health care needs during study procedures

At every study measurement visit, any individual found to have an unmet health need (whether physical or mental) will be referred to the relevant service within the Gugulethu CHC or at higher levels of care, as appropriate. In particular, any individual found to be non-adherent to ART during the study will be referred to clinic staff for adherence counselling and then to the relevant ART service as per randomization assignment.

Staff Training

Prior to the initiation of the study, all staff who will have contact with participants will take part in a study-specific training. The curriculum of the training will include: rationale, purpose, and scientific objectives of the study; study design and methodology; conduct of study assessments, tracking of participants, completion of study forms, and data collection; staff responsibilities; recruiting participants; procedures for enrolling participants into the study; randomization, universal precautions, communication skills, safety in the field, ethical guidelines for research including participants’ rights; procedures for obtaining informed consent; and confidentiality requirements. There will also be training on adherence assessment and referral for those participants admitting poor adherence.

Study staff will receive hands-on training that will include an introduction to data collection forms and procedures. Mock interviews will be an essential component of the training and protocol team members will act as both the trainer and the mock respondent. The trainer will take the staff through each step of the data collection process, from enrolling participants to ending the study visit and completing the necessary forms. Study staff will be given a chance to practice both the English and isiXhosa versions of all the assessments in order to discuss and resolve any issues. Training for study staff is expected to take approximately 1 week with additional follow-up training, as necessary.

All staff who, through the course of their work, will have knowledge of, or access to, personal information about participants will be required to complete training on patient confidentiality and sign a confidentiality agreement before the start of data collection.

Study staff members who will collect blood samples and conduct laboratory analyses will receive training in universal precautions, sample collection, testing of study samples and on the SOPs in phlebotomy and DBS.

In addition, staff will be trained in the management of crisis situations, including reports of abuse and domestic violence that may be disclosed during study participation. The site will have established procedures for managing these situations including counselling and appropriate referrals. Procedures for managing these situations will be outlined in study SOPs.

For all study staff, there will be additional training days scheduled during the study for refresher training. During these refresher trainings, study staff will review study procedures and discuss any challenges encountered

Contamination and masking

Contamination between groups is a concern but blinding participants to this type of health systems evaluation is not possible. Contamination in this case means that participants in the delayed referral group may be aware of the early referral group participants who are already in the adherence club system, and vice versa, so potentially changing their behaviours related to their own ART use and retention in care

Detection biases are possible for self-reported outcomes due to lack of masking, but the primary outcome (a suppressed viral load) is objective. We will conduct the study measurement visits from a space at the Gugulethu CHC that is separate from both the general adult ART services and the adherence club service, with study staff who are different from participants’ service provider, in order to minimize possible biases in assessment. We will also blind data analysis by the coding of study arm until the main trial analysis is complete and approved by the investigator team.

**8. Participant Retention**

At enrolment, all participants will be asked to provide detailed locator information. Specifically, participants will be asked to provide their full name, address and contact numbers (including landline and cellphone). In addition, the names, addresses and contact details of two individuals with whom the participant lives and one individual with whom the participant does not live will be requested. Participants will be asked to update their locator information at all subsequent visits as necessary. All locator information will be kept in locked study cabinets accessible only under the direction of the study coordinator.

After enrolment, all study activities will take place through appointments conducted separately from the ART care provided at the clinic or clubs. All participants will receive reminder calls one day prior to their scheduled study visit.

There will be two main approaches to tracing participants who miss study appointments:

- Telephonic contact with the participant or with an alternate individual pre-designated by the participant will be attempted.
- If telephone attempts fail, study fieldworkers will visit the home address of the participant to trace the participant in person.

All participant tracing efforts by any staff (via phone or home visit) will not mention the reason for the contact, or anything regarding HIV/AIDS or ART. All contacts simply request that the participant come to the clinic the next working day for a health-related issue. Our research team has been working in this setting for more than 8 years, has close knowledge of the local community, and has extensive experience with retention activities around HIV/AIDS.

As part of research activities, we will include an assessment of whether the participant is taking ART. For those individuals found not to be using ART, staff will facilitate an urgent referral back to the appropriate services as per randomization assignment. Details and examples of these retention procedures, including the adherence assessment, will be included in the staff training to be conducted prior to study implementation.

Participant withdrawal

All participants may refuse or voluntarily withdraw from the study for any reason and at any time. As part of the informed consent process, staff will state specifically that participation in the study is voluntary and that a participant may refuse participation or withdraw from study participation at any time. Participants will be told that withdrawal from the study will have no effect on their access to health facilities or HIV-related testing, care and treatment services. All study staff will be trained to ensure that participants have a firm understanding of this concept at the time of the informed consent process; per consenting requirements, the informed consent form will also include a statement to this effect.

**9. Analytic considerations**

Sample size considerations

The projected sample size for the trial is 214 participants

This estimate is based on the following assumptions:

- A superiority comparison of the immediate and delayed club referrals.
- 90% power and a two-sided alpha of 0.05 using a log-rank test of survival proportions
- 1:1 randomization
- An expected proportion of virally suppressed adults in the delayed referral group of 65% (our best estimate of this is 68% from data collected at Gugulethu) and an expected proportion of adults in the early referral group of 85%. Thus the expected minimum difference in the primary outcome at 12 months post-initiation between the early and delayed arms is 20%.
- Furthermore, the proposed sample size will also provide >80% power to detect appreciable differences for most secondary outcomes of interest.

Data management

Data management will be as for the PACART study based at UCT and Gugulethu. Data collected on paper forms will be entered into a custom designed Microsoft Access database, maintained in a firewall-protected UCT server with nightly backups. The study database will be password-protected following standard password safety procedures. The database will be designed and maintained by a senior data manager who will develop the data dictionary, direct queries, and data quality assurance / quality control activities, and will supervise the data entry clerk. Data quality assurance will be in the form of robust database structure and platform, “front-end” data checks, including real-time database queries. Quality control will be through data checking scripts to identify out-of-range values, logic violations, and missing observations. Data editing will be based on reference to the form and/or source document in question; all data queries and responses will be logged, and edits will be implemented through separate program files. All study records will contain anonymous participant identification numbers, and no participant names or identifiers will be recorded.

Data analysis

Data will be exported to Stata Version 13.0 (Stata Corporation, College Station, Texas) or R (Gnu Project) for analysis. The full statistical analysis plan will be developed by the trial statistician as per the PACART trial. Here we provide a brief outline of planned analyses.

For the **primary objective**, we hypothesize that adults randomized to early club referral will be more likely to maintain viral suppression (defined as <400 copies/ml, with secondary analyses at other thresholds) up to 12 months post-ART initiation, compared to adults randomized to delayed club referral.

Comparisons of study arms at enrolment will examine demographic, clinical and relevant behavioural variables. Primary analyses will be by intention-to-treat in participants with a VL <400 copies/mL. Secondary ‘per protocol’ analyses will be based on actual attendance at least one visit at the intended service. Additive binomial regression models will be used to assess the effect of trial arm on the primary outcome, with results presented as absolute risk differences with 95% confidence intervals. Models will be adjusted for clinical and demographic characteristics thought to predict the outcome while not mediating the intervention effect. Intervention effects will be examined across *a priori* subgroups including age, gender, previous antiretroviral exposure and nadir CD4 cell count.

For the **secondary objectives**, we hypothesize that adults randomized to early club referral will be more likely to be retained in care (defined as attending ART clinical appointments as scheduled), compared to adults randomised to delayed club referral. Analyses of other secondary outcomes (including measures of health status and health service utilization, as well as quantitative acceptability measures) will be based on pure-count methods, beginning with a description of variables overall and by arm using proportions with exact 95% confidence intervals, or median with interquartile ranges. Comparisons of distributions by trial arm will employ exact tests or rank-sum tests, as appropriate. Throughout statistical tests will be 2-sided at α=0.05, with the exception of primary outcome analyses that will be adjusted for interim looks (see below). Generalized linear models will be used to examine multivariable prediction of outcomes of interest. Models with repeated outcomes per subject over time will account for within-subject correlation through subject-specific random effects. Model fit will be compared using AIC and LR statistics; all modelling will employ standard diagnostic procedures.

Interim analyses

The Data Monitoring Committee will monitor trial progress and conduct periodic reviews of the trial outcomes. This independent review will be valuable in maximizing the benefits of the trial findings for scientific and policymaking purposes. The DMC will review: (i) the rate of recruitment; (ii) any adverse events identified during the study including social adverse events; and (iii) evidence for the primary outcome (viral suppression) including evidence for superiority or inferiority. On advice from the Trial Steering Committee, we propose that stopping rules be provided to the DMC only as guidelines to help assist in any decision making around stopping the trial due to evidence of inferiority or superiority in the primary outcome

**10. Ethical considerations**

Ethical review

The study protocol, informed consent forms, all data collection tools, participant materials and other requested documents will be submitted to Human Subjects Research Ethics Committee of the Faculty of Health Sciences at the University of Cape Town.

Informed consent

Informed consent before enrolment will be delivered in participants’ home language (isiXhosa) by trained interviewers following a standardized script. This script details the purpose of the study, the nature of randomization, study procedures throughout the trial period, and the risks and benefits that participants may encounter during the study. Here and throughout the study, study staff will emphasize to participants that:

- Participation is entirely voluntary, and their choice regarding participation will in no way influence the quality of their routine medical care.
- Individuals may exit the study at any time for any reason without compromising the quality of health care received.

English versions of the informed consent documents are provided in Appendix A. Translated isiXhosa versions (as well as a certification of their translation and back translation) will be lodged with the IRB/REC before the start of the study.

Risks

The trial comparison is of timing of referral to the adult ART adherence club service – early or delayed. The antiretroviral medications used are identical between the two arms, and thus there is minimal biomedical risk related to participation in this regard. There remains clinical equipoise regarding the benefit of adherence club referral in this population and so the ethical principles of beneficience and justice are upheld. Regarding any potential risks of poorer outcomes for the delayed referral arm, the DMC will advise as above.

In addition to phlebotomy done as part of routine care, blood samples are drawn at each study measurement visit. To minimize the accompanying risks of complications of phlebotomy, blood will be drawn by experienced staff under sterile conditions, and with the guidance of a phlebotomy SOP.

There are potential psychosocial risks which are commonly encountered in this type of research.

**First**, study assessments include measures of behavioural and psychosocial information including disclosure, stigma, social support and related issues, as these are important risk factors for the outcome of interest and may modify the impact of the intervention. There is some risk that participants may experience psychological distress during these assessments, and to address this, our interviewers will be trained in appropriate interviewing techniques, recognition of signs of psychosocial distress of different types, and referral resources and procedures for these.

**Second**, it is possible that allocation to either arm could result in psychological distress related to being seen to attend an HIV-related health care service, in a setting where stigmatization of HIV infection persists. In addition to the procedures outlined above to detect, contain and refer such cases, we will measure perceived stigma as part of assessments.

**Third**, there are potential risks should loss of confidentiality occur during study procedures—for instance, in the process of data collection or participant follow-up. Specific measures to minimize loss of confidentiality are detailed in the confidentiality section below.

**Lastly**, although it does not constitute a risk related to participation, being found to have an unsuppressed viral load is a negative health outcome associated with morbidity and mortality. Any patient having been found to have an unsuppressed viral load through study measurement procedures will be counselled appropriately and referred to their respective care service for further management through the public sector.

All participants will be informed of these risks, and the strategies to minimize these, as part of the informed consent process. The alternative to participation is clinic care with possible club referral from 6 months post-initiation if virally suppressed. Therefore, each of these classes of risk are encountered during routine health care services. However, the study team has integrated specific steps in the study design and conduct to minimize the possibility of these risks. These steps draw directly from prior experiences conducting research on HIV prevention and treatment in Gugulethu and similar communities across Cape Town.

Benefits

*Direct benefit*

The major potential direct benefit from participating in this study is optimized ART use. This, in turn, is likely to maximize the individual’s physical health. An optimal ART service is also likely to have social benefits such as reduced time waiting for medication dispensing and increased compatibility with work commitments.

*Indirect benefit*

By identifying the optimal strategy for delivering ART to newly initiated adults, this study has the potential to lead to improved ART services in Cape Town, the Western Cape Province, and across South Africa. To this end, the involvement of policy makers involved in HIV care and treatment will help maximize the indirect benefits of the study through strengthened public sector health care services for HIV-infected individuals.

Compensation

At each visit, participants will be given up to R20 in cash to cover the cost of transport to their next scheduled study measurement visit, R120 in the form of grocery vouchers and a maximum of R50 worth of refreshments. Note that no compensation or reimbursement in any form will be provided to adults attending routine care and/or ART-related visits at any time during the study.

Confidentiality

The following steps will be taken to minimize the risk of any loss of confidentiality throughout study design and conduct:

- All personnel involved in data collection and management will undergo study-specific training in confidentiality and related patient protection issues.
- Following standard practice, all patient- and study-related information will be kept in locked cabinets at either the study office in Gugulethu or at UCT.
- Anonymous participant identification numbers will be used on all study documents. Collection of participant names and other identifiers will be restricted to informed consent documents, patient tracing materials, and a study identification key, all of which will be kept in a locked cabinet in the study office at Gugulethu and at UCT separate from other study documentation and accessible only by the project coordinator and local PI. No CRF will include participant name.
- All electronic records will be kept in password-protected files. All electronic communications of study data will be through password-protected, encrypted files. All data storage at the University of Cape Town will be within a firewall-protected SQL server. While efforts will be made to minimize the loss of confidentiality, in the event that staff learn that the participant is a threat to themselves or to others, the proper authorities will be notified. This exception will be included in all study informed consent forms.

Internal monitoring

Throughout the conduct of the study, internal study monitoring will be led by the study PIs. Study PIs, co-investigators and the study coordinator will participate in weekly meetings to monitor the rate of participant enrolment and the integrity of protocol implementation (including the completion of informed consent and quality of study measures). In addition, participant retention and safety endpoints will be discussed. In addition to these internal reviews, any individual found to have defaulted ART during the study will be re-referred to the relevant ART service (clinic or club) after intensive counselling on ART use.

Use of information and publications

Publication or presentation of the results of this study will be agreed upon in collaboration with the study investigators. Note that the funding agency has no input in the decision to present or publish study data or the nature of the data that are presented or published.

Appendix A

Informed Consent Forms 1 and 2

**11. References**

1. UNAIDS. 90–90–90 - An ambitious treatment target to help end the AIDS epidemic | UNAIDS. In: *UNAIDS*. Geneva, Switzerland; 2014. http://www.unaids.org/en/resources/documents/2014/90-90-90. Accessed April 25, 2016.

2. MacLeod W, Fraser N, Shubber Z, Carmona S, Pillay Y, Gorgens M. Analysis of age-and sex-specific HIV care cascades in South Africa suggests unequal progress towards UNAIDS 90-90-90 targets. *21st International AIDS Conference, Durban*, 2016.

3. Fox M, Rosen S. Retention of adult patients on antiretroviral therapy in low- and middle-income countries: systematic review and meta-analysis 2008-2013. *J Acquir Immune Defic Syndr*. 2015;69:98-108. doi:10.1161/CIRCRESAHA.116.303790.The.

4. Dewing S, Mathews C, Cloete A, et al. From research to practice: Lay adherence counsellors’ fidelity to an evidence-based intervention for promoting adherence to antiretroviral treatment in the Western Cape, South Africa. *AIDS Behav*. 2013;17(9):2935-2945. doi:10.1007/s10461-013-0509-x.

5. Luque-Fernandez MA, Van Cutsem G, Goemaere E, et al. Effectiveness of Patient Adherence Groups as a Model of Care for Stable Patients on Antiretroviral Therapy in Khayelitsha, Cape Town, South Africa. *PLoS One*. 2013;8(2). doi:10.1371/journal.pone.0056088.

6. Wilkinson LS. ART adherence clubs: A long-term retention strategy for clinically stable patients receiving antiretroviral therapy. *South Afr J HIV Med*. 2013;14(2):48-50. doi:10.4102/hivmed.v14i2.77.

7. Solomon S, et al. Community based ART adherence clubs: A community model of care for ART delivery. *7th South African AIDS Conference, June 2015, Durban, South Africa.*

8. Fatti G, Meintjies G, Shea J, Eley B, Grimwood A. Improved survival and antiretroviral treatment outcomes in adults receiving community-based adherence support: 5-year results from a multicentre cohort study in South Africa. *J Acquir Immune Defic Syndr*. 2012;61(4):e50-e58.

9. Nachega J, Adetokunboh O, Uthman O, et al. Community-Based Interventions to Improve and Sustain Antiretroviral Therapy Adherence, Retention in HIV Care and Clinical Outcomes in Low- and Middle-Income Countries for Achieving the UNAIDS 90-90-90 Targets. *Curr HIV/AIDS Rep*. 2016;13(5):241-255.

10. Grimsrud A, Sharp J, Kalombo C, Bekker L-G, Myer L. Implementation of community-based adherence clubs for stable antiretroviral therapy patients in Cape Town, South Africa. *J Int AIDS Soc*. 2015;18(1):19984. doi:10.7448/ias.18.1.19984.

11. Grimsrud A, Lesosky M, Kalombo C, Bekker L-G, Myer L. Community-Based Adherence Clubs for the Management of Stable Antiretroviral Therapy Patients in Cape Town, South Africa: A Cohort Study. *J Acquir Immune Defic Syndr*. 2016;71:e16-e23.

12. Bango F, Ashmore J, Wilkinson L, van Cutsem G, Cleary S. Adherence clubs for long-term provision of antiretroviral therapy: cost-effectiveness and access analysis from Khayelitsha, South Africa. *Trop Med Int Health*. 2016;21(9):1115-1123.

Appendix A: Informed Consent Form 1_v3 and Informed Consent Form 2_v1

Informed Consent Form 1

**Title of Research: Timing of Adherence Club Referral (TRAC)**

**WHAT IS THE PURPOSE OF THE STUDY?**

We are from the University of Cape Town. We are doing a research study to compare two different ways of providing HIV treatment to adults. You are being asked to take part in this study, which is being done at the Gugulethu Community Health Centre (CHC).

In this study we are comparing two different ways of providing this treatment. Information learned in this study will help us to improve adult HIV services, so that they can remain in care and adherent to treatment.

You are being asked to take part in this study because you are an adult with known HIV-infection who started taking HIV drugs 4-5 months ago (ART, anti-retroviral therapy), and who is adherent to them. The purpose of this consent form is to give you information to help you decide if you want to take part in this study or not.

**WHAT HAPPENS IF I AGREE TO TAKE PART?**

**Enrolment and randomization**

If you agree to take part you will do the following today:

- Answer questions about a number of topics including: your household, your contact details, your medical history, your HIV testing history and your use of HIV medications.
- Have 5mls (1 teaspoon) of blood taken from your arm.
- You will then be randomized (like a flip of a coin) to one of two groups, who will each receive their HIV treatment in different ways:

1. **Community-based adherence club:** Adults assigned to this group will receive HIV care and medicines at the adherence clubs which meet at the community centre about 600m from the Gugulethu Community Health Centre (CHC). They will be referred to the clubs straight away at enrolment.
2. **General antiretroviral therapy (ART) clinic:** Adults assigned to this group will be referred to the ART clinic at the Gugulethu CHC for HIV care and treatment. They will only be referred to clubs, by the clinic, after they have been on ART for at least 12 months.

**“Randomized”** means that you will have a 50% chance of being in the group that will receive care at the adherence club. You will also have a 50% chance of being in the group that gets referred to an ART clinic. Neither the study staff nor you can choose which group you will be assigned to. The decisions are made by a computer and put into an envelope. The staff does not know which group is in each envelope.

**Study measurements visits**

After you are randomized, you be asked to attend a further 2 study measurement visits at approximately 4 months and 8 months post-enrolment. These study visits are separate from the usual clinic visits that you will have for your HIV care. Each visit will take about 60 minutes.

At these visits, you will do the following:

- Answer different sets of questions at each visit. Questions may include the following topics: your recent medical history, your HIV medications, HIV disclosure, stigma, physical and mental health (including drug and alcohol use), and how you feel about the HIV care that you have received.
- Have 5mLs (1 teaspoon) of blood drawn from your arm at every visit.

Your last study measurement visit will be at 8 months after enrolment. After this you will no longer be required to attend study visits, but will continue with routine HIV care either at the adherence club or at the ART clinic as appropriate.

**NOTE:** The blood that is drawn at each visit will be stored and used to check your viral load (this is the amount of HIV in your blood) at a later time. Results from these tests will not be available to you, the clinic, or the study staff. Your stored blood and any study test results will not have your name or any other means of identifying you on it. When the health care workers who do your routine follow-ups at the clinic or the adherence club need to check your viral load, they will take a separate blood specimen.

**REVIEW OF MEDICAL RECORDS**

As part of this study we will be looking at and taking information from your laboratory and pharmacy records as well as your ART clinic or adherence club records. From these records we are interested in your HIV care. All the data that we review and abstract is confidential and no participant names are recorded on study documents.

As part of this study, we will also look at your health records at government clinics and hospitals within the Western Cape for up to 3 years after your study enrolment.

All the information used in this study will be treated as highly confidential and will be securely stored. For the study analysis, no patient names or identifying information will be available to the researchers.

**FOLLOW-UP OF MISSED VISITS**

You will be asked to provide contact information so that we can get in touch with you during the study. Study staff will talk to you about the best way to contact you. If you miss one of the scheduled study visits, a member of the study staff will contact you in order to find another day and time to complete your visit. If you repeatedly miss study visits or the staff is unable to contact you using the information that you provide, it may be necessary to visit you at home in order to reschedule the missed study visit.

**CONTACT FOR FUTURE STUDY**

After the completion of your last visit at 8 months after enrolment, we might contact you again in the future to take part in other research studies. At that time, you would be asked to review and sign another consent form. You can choose not to take part in any future studies if you are asked. You will be asked to provide contact information so that we can get in touch with you regarding additional research studies. Study staff will talk with you about the best way to contact you.

**WHAT ARE THE POTENTIAL RISKS OF TAKING PART IN THE STUDY?**

You may feel uncomfortable about some of the personal questions you are asked. You may refuse to answer any question that you do not want to answer.

There is some risk in sharing personal and medical information. We will be careful to keep all your information as private as possible.

Drawing blood is normally done as part of routine medical care and presents a slight risk of discomfort. Experienced staff will draw blood under sterile conditions in order to protect you against these risks.

**WHAT ARE THE POTENTIAL BENEFITS OF TAKING PART IN THE STUDY?**

There is no direct benefit to you if you take part in this study, but if we identify any health care problem for you during the course of the study, we will make sure that you are referred to the appropriate health care services. In addition, the information gained in this study may help to improve adult ART services for HIV-infected adults in Cape Town, the Western Cape Province, and across South Africa.

**WHAT ARE THE ALTERNATIVES TO TAKING PART?**

The alternative to taking part in this study is to continue with the standard of care for all HIV-positive adults on ART at Gugulethu CHC which means that you will continue to attend the ART clinic. As part of the standard of care, if you are taking your ART pills every day and your blood tests show that your HIV is under control, you will be considered for club referral by the clinic staff when you have been on ART for at least 6 months.

**WHAT ABOUT CONFIDENTIALITY?**

If you agree to take part, all information collected during the study will be kept strictly confidential. Your name will not be written on the study forms and it will not be used in connection with any information or lab specimens that are collected as part of the study.

All study materials will be stored in locked filing cabinets. Only study staff and personnel involved in routine audits will have access to these materials. All staff involved in data collection and management will get specific training in confidentiality.

While these efforts will be made to maintain confidentiality, if the study staff learns that you are a risk to yourself or someone else, they will tell the proper authorities.

**WHAT ABOUT INSURANCE?**

There are no experimental medicines being used in this study. Therefore, no insurance has been obtained. However, you will be protected in terms of the study staffs’ personal malpractice insurance or the university’s insurance cover in the event of injury or illness that is caused by you taking part in this study (details of this insurance cover are attached in the appendix at the end of this document).

**WILL I BE GIVEN ANYTHING FOR TAKING PART?**

At the end of each visit, you will be given R20 in cash to cover the transport cost to your next scheduled study visit and an R120 grocery voucher.. Refreshments will be provided at all visits.

**ARE THERE ANY COSTS TO TAKING PART IN THE STUDY?**

There is no cost for being in this study.

**CAN I LEAVE THE STUDY?**

You have the right to decide not to take part in the study, to refuse to answer any questions, or to withdraw from the study at any time without penalty. It will have no effect on the care that you receive at the Gugulethu CHC or any other health facility.

**DO YOU HAVE ANY QUESTIONS?**

If there is anything that is unclear or if you need further information, please ask us and we will provide it. Do you have any questions currently?

**ADDITIONAL INFORMATION:**

If you have any questions or have any problems while taking part in this research study, you should contact:

Prof Landon Myer

School of Public Health and Family Medicine

Faculty of Health Sciences, University of Cape Town

Tel: 021 406 6661

Email: [Landon.Myer@uct.ac.za](mailto:Landon.Myer@uct.ac.za)

If you have any questions about your rights as a research participant, you may contact the following member of the ethics committee:

Prof Marc Blockman

Chair, Human Research Ethics Committee

Faculty of Health Sciences, University of Cape Town

Tel: 021 406 6338

**CONSENT STATEMENT:**

I have read this form, or someone has read it to me. I have been offered a copy of this consent form. I was encouraged and given time to ask questions. I agree to be in this study. I know that after choosing to be in this study, I may withdraw at any time. My being in the study is voluntary. I understand that whether or not I participate will not affect my health care services received today, or at any time in the future.

***PLEASE INDICATE YOUR CONSENT BELOW WITH YOUR SIGNATURE.***

***Volunteer*:**

*Name* *and Surname _____________________________________________________*

*Signature* __________________________ *Date* ________________________

***Staff member:***

*Name* *and Surname _____________________________________________________*

*Signature* __________________________ *Date* ________________________

***IF THE VOLUNTEER IS UNABLE TO READ OR WRITE, THE VOLUNTEER MUST INDICATE CONSENT WITH A FINGERPRINT AND THE ENTIRE COUNSELLING PROCESS MUST BE OBSERVED BY AN INDEPENDENT WITNESS WHO CAN CONFIRM THE PROCEDURE ONCE CONSENT HAS BEEN GIVEN.***

***Fingerprint of volunteer:***

***Witness*:**

*I confirm that I am independent of the study and that I witnessed the entire informed consent counselling process in the home language of the volunteer*

*Name* *and Surname _____________________________________________________*

*Signature* __________________________ *Date* ________________________

***THANK YOU***

Informed Consent Form 2

**Title of Research: Timing of Adherence Club Referral (TRAC)**

**CONSENT FOR STORAGE AND FUTURE USE OF UNSTORED SPECIMENS**

**WHAT IS THE PURPOSE OF THIS DOCUMENT?**

As part of being in the TRAC study, you will have blood drawn to check your viral load (this is the amount of HIV in your blood) at each visit. There may be leftover blood from the samples that you provide. This document will provide you with information about the storage of these leftover blood samples. After we talk about this, we will ask you to sign a consent form to state whether you are willing or unwilling to allow us to store any leftover blood collected from you as part of the TRAC study. You will be allowed to continue to take part in the TRAC study whether you agree to store these leftover bloods or not.

**WHAT WILL HAPPEN IF I AGREE TO HAVE MY LEFTOVER BLOOD STORED?**

If you agree, any leftover blood from the samples you provided as part of this research project may be stored and used for future HIV related research.

**HOW WILL THE LEFTOVER BLOOD BE USED?**

It is possible that these stored samples may be tested to see if the HIV in your blood is resistant to any types of HIV medications or to look at other questions related to HIV. It is also possible that the stored blood may be used to look at other questions related to general health.

At this time, we cannot provide details of when this additional testing may be conducted. However, additional testing will not be done using these stored samples without the approval of the appropriate ethics committees involved in this research.

**WHAT ARE THE RISKS AND BENEFITS OF STORING LEFTOVER BLOOD?**

There is no direct benefit to you if you allow us to store your specimens for future research. The information gained from additional study of the stored specimens may help to improve our understanding of HIV and ART (anti-retroviral therapy) and may help to improve adult ART services in Cape Town, the Western Cape Province, and across South Africa. There are no risks to your health related to storing your samples other than those associated with blood collection as described in the main informed consent document.

**WHAT ABOUT CONFIDENTIALITY?**

If you agree to have specimens stored, all information collected will be kept strictly confidential and all staff involved in data collection and management will get specific training on confidentiality. The specimens will only have your study number on them. Neither your name nor other identifiers will be included with any information resulting from use of these stored specimens. The results of additional tests will not be linked to you and you will not be informed of these test results.

**HOW LONG WILL THESE SAMPLES BE STORED?**

If you agree to let us store your leftover samples for future research, they will be kept in a locked freezer for up to 5 years.

**CAN I REFUSE TO STORE LEFTOVER BLOOD?**

Some people will not agree to have their specimens used for future research and you are free to choose if you do not want your specimens to be stored for research other than related to this study, or not be used at all. This decision will not affect your participation in the current study in any way, nor will it influence the care you receive here at the Gugulethu CHC or any health care clinic.

If you agree today, you may also withdraw permission at any time. You can do so here with the study team or you can contact the researcher below:

Prof Landon Myer

School of Public Health and Family Medicine

Faculty of Health Sciences, University of Cape Town

Tel: 021 406 6661

Email: [Landon.Myer@uct.ac.za](mailto:Landon.Myer@uct.ac.za)

If you have any questions about your rights as a research participant, you may contact the following member of the ethics committee:

Prof Marc Blockman

Human Research Ethics Committee

University of Cape Town

**Tel: 021 406 06338**

**CONSENT STATEMENT:**

**Choose one of the 3 options below by initialling on the relevant line:**

If any of the blood I have provided for this research project is unused or left over when the research is completed:

1. ______ I do NOT agree to the storage of my blood for future use.
2. ______ I agree to have my blood stored for future research relevant to HIV health

approved by a registered human research ethics committee

1. ______ I agree to have my blood stored for future research related to this study ONLY

I have read this form, or someone has read it to me. I have been offered a copy of this consent form. I was encouraged and given time to ask questions. I agree to be in this study. I know that after choosing to be in this study, I may withdraw at any time. My being in the study is voluntary. I understand that whether or not I participate will not affect my health care services received today, or at any time in the future.

PLEASE INDICATE YOUR CONSENT WITH YOUR SIGNATURE:

**Volunteer:**

Name and Surname _____________________________________________________

Signature _____________________ Date ________________________

**Staff member:**

Name and Surname_____________________________________________________

Signature _____________________ Date ________________________

IF THE VOLUNTEER IS UNABLE TO READ OR WRITE, THE VOLUNTEER MUST INDICATE CONSENT WITH A FINGERPRINT AND THE ENTIRE COUNSELLING PROCESS MUST BE OBSERVED BY AN INDEPENDENT WITNESS WHO CAN CONFIRM THE PROCEDURE ONCE CONSENT HAS BEEN GIVEN.

**Fingerprint of volunteer:**

**Witness:**

I confirm that I am independent of the study and that I witnessed the entire informed consent counselling process in the home language of the volunteer

Name and Surname_____________________________________________________

Signature _____________________ Date ________________________

**THANK YOU**
